# The endothelial transcription factor ERG mediates Angiopoietin-1-dependent control of Notch signalling and vascular stability

**DOI:** 10.1038/ncomms16002

**Published:** 2017-07-11

**Authors:** A. V. Shah, G. M. Birdsey, C. Peghaire, M. E. Pitulescu, N. P. Dufton, Y. Yang, I. Weinberg, L. Osuna Almagro, L. Payne, J. C. Mason, H. Gerhardt, R. H. Adams, A. M. Randi

**Affiliations:** 1 Vascular Sciences, Imperial Centre for Translational and Experimental Medicine, National Heart and Lung Institute, Imperial College London, London W12 0NN, UK; 2 Department of Tissue Morphogenesis, Max Planck Institute for Molecular Biomedicine, Faculty of Medicine, University of Münster, D-48149 Münster, Germany; 3 Vascular Patterning Laboratory, Vesalius Research Center, VIB, KU Leuven, 3000 Leuven, Belgium & Max-Delbrück-Center for Molecular Medicine, Berlin 13125, Germany

## Abstract

Notch and Angiopoietin-1 (Ang1)/Tie2 pathways are crucial for vascular maturation and stability. Here we identify the transcription factor ERG as a key regulator of endothelial Notch signalling. We show that ERG controls the balance between Notch ligands by driving Delta-like ligand 4 (Dll4) while repressing Jagged1 (Jag1) expression. *In vivo*, this regulation occurs selectively in the maturing plexus of the mouse developing retina, where Ang1/Tie2 signalling is active. We find that ERG mediates Ang1-dependent regulation of Notch ligands and is required for the stabilizing effects of Ang1 *in vivo*. We show that Ang1 induces ERG phosphorylation in a phosphoinositide 3-kinase (PI3K)/Akt-dependent manner, resulting in ERG enrichment at Dll4 promoter and multiple enhancers. Finally, we demonstrate that ERG directly interacts with Notch intracellular domain (NICD) and β-catenin and is required for Ang1-dependent β-catenin recruitment at the Dll4 locus. We propose that ERG coordinates Ang1, β-catenin and Notch signalling to promote vascular stability.

## Abstract (web summary)

Vascular maturation and stability is regulated by Notch and Angiopoietin-1/Tie2 signalling. Here, Shah *et al*. show that the transcription factor ERG coordinates the Ang1, Notch and Wnt/β-catenin pathways to promote vascular maturation and stability.

Notch signalling is essential for the establishment, maturation and maintenance of a functional vascular network^1^,^2^. Notch regulates multiple aspects of vascular development including arterial/venous determination, tip/stalk cell specification, nascent vessel maturation and stability. Distinct pathways can selectively activate Notch in the different phases of vascular development and during maintenance. While insight into the regulation of Notch signalling in determining tip-stalk cell identity and endothelial sprouting is rapidly increasing, the transcriptional control of Notch signalling during vascular maturation remains poorly understood.

Of the four Notch ligands expressed in the endothelium in vertebrates, only loss of Delta-like ligand 4 (Dll4) or Jagged1 (Jag1) results in vascular defects^3^. The phenotypes exhibited by Jag1 (ref. 4) and Dll4 (refs 5, 6, 7) global knockout mice suggest that these two ligands are not functionally redundant. Indeed, Dll4 and Jag1 appear to play opposite roles in mouse retinal angiogenesis, since inhibition of angiogenesis by Dll4 can be competitively opposed by Jag1 (ref. 8). These two Notch ligands also show distinct spatial expression patterns in the postnatal retina vasculature, with Dll4 highly expressed in tip cells and also present at the edge of the growing plexus, while Jag1 expression is low/absent in tips cells but higher in adjacent stalk cells^8^. The equilibrium between these two Notch ligands is required for the formation of fully functional and stable vascular networks. Aside from its role in tip-stalk cell communication, Dll4/Notch signalling is also critical in the maturing vascular plexus at tight inter-endothelial cell–cell contacts; here Dll4/Notch signalling has been shown to negatively regulate blood vessel growth, promoting endothelial quiescence and vascular stability^9^,^10^,^11^.

The Angiopoietin-1 (Ang1)/Tie2 system plays an essential role in the maturation of nascent blood vessels and in maintaining vascular integrity by enhancing endothelial barrier function and promoting EC quiescence^12^,^13^,^14^,^15^. Several studies have shown that over-expression of Ang1 in mice leads to a stabilized, less permeable vasculature^16^,^17^, promoting endothelial survival and VE-cadherin-regulated inter-endothelial adhesion^15^. Interestingly, Zhang *et al*. showed that Ang1 upregulates Dll4 expression and Notch signalling *in vitro*, but only in the presence of cell–cell contacts, that is, in conditions that are supposed to mimic maturing or stable vasculature^18^. The authors also showed that Ang1-dependent potentiation of Dll4/Notch signalling required Akt-mediated activation of β-catenin, which formed a complex with NICD^18^, providing *in vitro* evidence for an integrated network of endothelial pathways promoting vascular maturation and stability.

We have recently identified a transcriptional pathway essential for vascular stability, coordinated by the endothelial transcription factor ERG (ETS-related gene). ERG is a member of the E-26 transformation specific (ETS) transcription factor family, which regulates a wide range of targets and pathways required for endothelial homeostasis, including proliferation, survival and barrier function^19^. Several *in vivo* and *in vitro* model systems have demonstrated the crucial role of ERG in vascular development, angiogenesis and vascular stability^19^,^20^,^21^,^22^,^23^,^24^,^25^,^26^. Mice deficient in endothelial ERG show severe defects in vascular development, resulting in embryonic lethality at E10.5–12.5 (refs 21, 24, 25). Inducible endothelial-specific deletion of ERG causes impairment in the retinal vasculature of newborn mice, with signs of vascular destabilization, such as loss of VE-cadherin expression, loss of pericyte coverage and increased number of empty collagen sleeves^21^. The vascular and molecular defects in the yolk sac of endothelial ERG-deficient mice were corrected by stabilization of β-catenin/Wnt signalling; *in vitro*, ERG was found to promote β-catenin protein stability through VE-cadherin and Wnt-dependent pathways^21^. Thus these studies showed that ERG promotes vascular stability through the Wnt/β-catenin signalling pathway.

In this study, we hypothesized that ERG acts to integrate the pathways described above to promote vascular maturation and stability. We used multiple approaches to show that ERG controls Notch signalling specifically during vascular remodelling and maturation, controlling the balance of expression between Dll4 and Jag1, two Notch ligands which act competitively. We show that ERG mediates the Ang1-dependent activation of Notch signalling in EC *in vitro* and *in vivo*. These results demonstrate that the transcription factor ERG coordinates the Ang1, Notch and Wnt/β-catenin pathways to promote vascular maturation and stability.

## Results

### ERG controls Notch signalling in differentiated EC

**Figure 1:**
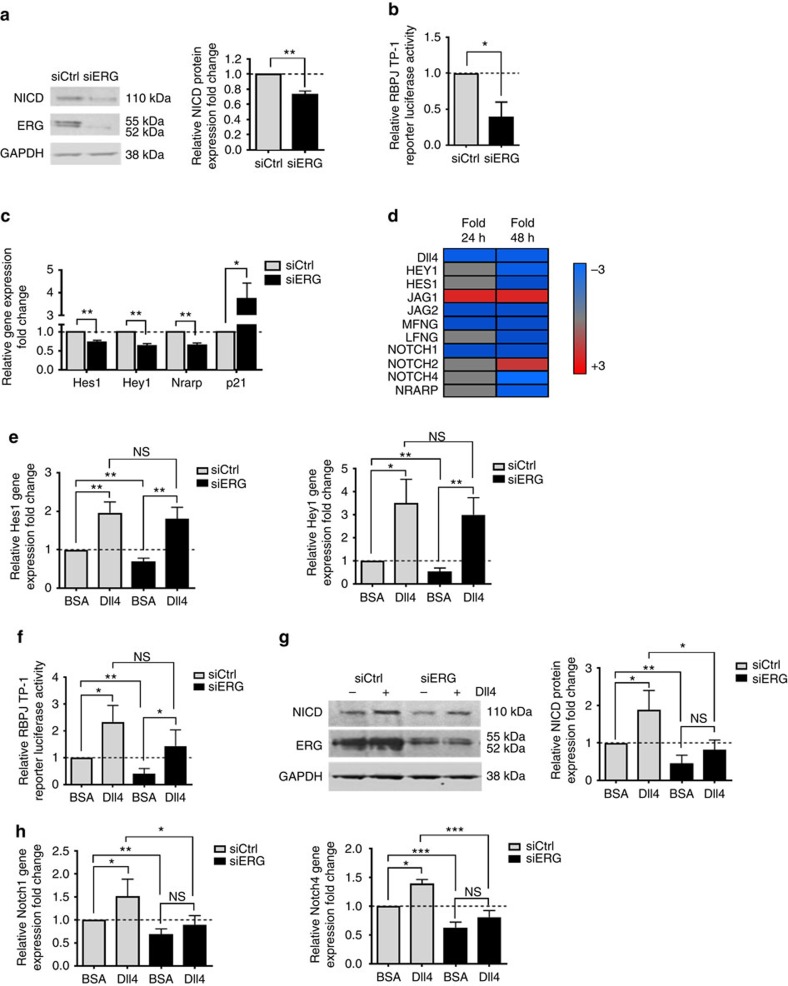
ERG regulates endothelial Notch signalling. (**a**) Western blot (WB) analysis of Notch intracellular domain (NICD) expression in control (siCtrl) and ERG-deficient (siERG) HUVEC (*n*=4). (**b**) RBP-J TP-1 Notch reporter activity in control and ERG-deficient HUVEC (*n*=4). (**c**) qPCR of Notch target gene expression in siCtrl and siERG-treated HUVEC: Hes1, Hey1, Nrarp and p21 (*n*=4). (**d**) Microarray and PCR screen analysis of differential gene expression in HUVEC was performed at 24 and 48 h after ERG inhibition^29^, with fold change of selected genes represented as high (red) and low (blue) expression compared to the median (grey). (**e**) qPCR analysis of Hes1 and Hey1 Notch target gene expression in siCtrl and siERG-transfected HUVEC stimulated with Dll4 or control BSA (*n*=4). (**f**) RBP-J TP-1 Notch reporter activity in control and ERG-deficient HUVEC plated on Dll4 or BSA (*n*=4). (**g**) WB analysis and quantification of NICD expression in siCtrl and siERG-transfected HUVEC stimulated with Dll4 or BSA (*n*=4). (**h**) qPCR analysis of Notch1 and Notch4 mRNA expression in siCtrl and siERG-transfected HUVEC stimulated with Dll4 or BSA (*n*=4). All graphical data are mean±s.e.m., *P<0.05, **P<0.01, ***P<0.001, Student’s *t*-test.

Ligand-mediated activation of Notch receptors promotes proteolytic release of the Notch intracellular domain (NICD), which then translocates to the nucleus and acts as a co-activator for the transcriptional regulator RBP-J^27^. Inhibition of ERG expression in HUVEC by ERG siRNA caused a decrease in NICD levels (Fig. 1a) and RBP-J luciferase reporter activity (Fig. 1b) compared to control siRNA. Consistently, ERG inhibition via two separate siRNA sequences downregulated expression of Notch downstream targets Hes1, Hey1 and Nrarp, while levels of p21CIP1 (p21) were increased (Fig. 1c and Supplementary Fig. 1a), in line with decreased Notch signalling^28^. The Notch pathway is controlled by a balance of activators and repressors. Inhibition of ERG expression in EC was found to affect multiple genes involved in the Notch pathway^29^ (Fig. 1d): ERG-deficient cells showed decreased levels of Notch receptors Notch1 and Notch4, but increased levels of Notch2 (Fig. 1d and Supplementary Fig. 1b). Levels of Notch modulators Manic Fringe and Lunatic Fringe (MFNG and LFNG) were also decreased (Fig. 1d and Supplementary Fig. 1c). In the absence of ERG, Dll4 stimulation was able to rescue expression of Notch targets Hes1 and Hey1 (Fig. 1e) and significantly increased RBPJ activity (Fig. 1f). Levels of NICD (Fig. 1g), Notch 1 and Notch 4 (Fig. 1h) were not normalized by stimulation with Dll4, in line with ERG’s direct transcriptional control of Notch receptors. (Supplementary Fig. 1d,e, respectively related to Fig. 1g,h, show ERG levels in these experiments). These data suggest that ERG controls Notch signalling at multiple levels.

### ERG controls the balance of expression between Dll4 and Jag1

**Figure 2:**
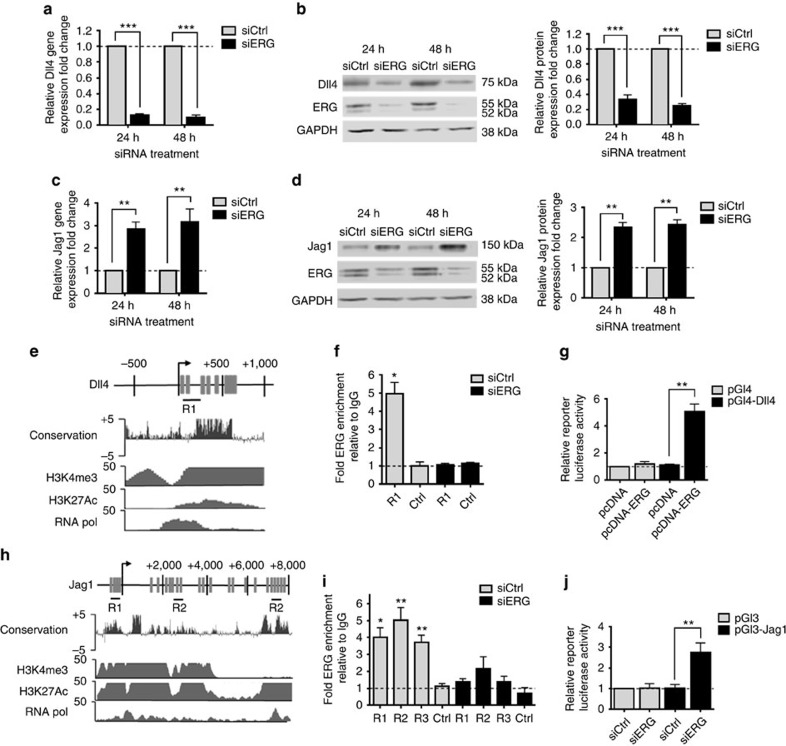
ERG transcriptionally activates Dll4 and represses Jag1 expression. (**a**) mRNA levels of Dll4 in HUVEC treated with siCtrl or siERG for 24 and 48 h (*n*=6). (**b**) Representative WB and quantification of Dll4 protein expression in siCtrl and siERG-treated HUVEC for 24 and 48 h (*n*=3). (**c**) mRNA levels of Jag1 in siCtrl and siERG-treated HUVEC for 24 and 48 h (*n*=6). (**d**) Representative WB and quantification of Jag1 expression in siCtrl and siERG-treated HUVEC for 24 and 48 h (*n*=6). (**e**) Putative ERG binding sites (grey bars) are located within the Dll4 promoter downstream of the transcription start site (TSS) (arrow); ENCODE sequence conservation between 100 vertebrates is shown across this region. ENCODE ChIP-seq data profiles for H3K4me3, H3K27Ac and RNA polymerase II (RNA pol) in HUVEC indicate open chromatin and active transcription. Location of qPCR amplicon covering region R1 is indicated. (**f**) ChIP-qPCR using primers to region R1 on ERG-bound chromatin from siCtrl or siERG-treated HUVEC. Primers for a region within exon11 of the Dll4 gene were used as negative control. Data are shown as fold change over IgG (*n*=3). (**g**) Dll4 promoter luciferase reporter assay. ERG cDNA expression plasmid (pcDNA-ERG) or empty expression plasmid (pcDNA) were co-transfected with a Dll4 promoter-luciferase construct (pGl4-Dll4, covering region R1) in HUVEC, and luciferase activity was measured. Values represent the fold change in relative luciferase activity over the empty pGL4 vector alone (*n*=4). (**h**) Putative ERG binding sites (grey bars) located within the Jag1 genomic locus. TSS is indicated (arrow); ENCODE sequence conservation between 100 vertebrates and ChIP-seq data profiles for H3K4me3, H3K27Ac and RNA polymerase II in HUVEC are shown across this region. Location of qPCR amplicons covering R1, R2 and R3 are indicated. (**i**) ChIP-qPCR using primers covering Jag1 promoter regions R1, R2, R3 and Ctrl region on ERG-bound chromatin from siCtrl or siERG HUVEC (*n*=4). (**j**) Control or Jag1 promoter luciferase construct (pGl3-Jag1, covering regions R1 and R2) activity after siERG treatment. Results are expressed as luciferase activity relative to siCtrl-treated cells (*n*=3). All graphical data are mean±s.e.m., *P<0.05, **P<0.01, ***P<0.001, Student’s *t*-test.

The Notch ligands Dll4 and Jag1 have been shown to exert opposite effects on vascular development and angiogenesis^8^,^30^. Interestingly, inhibition of ERG expression resulted in a decrease in Dll4 mRNA (Fig. 2a and Supplementary Fig. 1f) and protein expression (Fig. 2b) while causing an increase in Jag1 mRNA (Fig. 2c and Supplementary Fig. 1f) and protein levels (Fig. 2d), suggesting an inverse regulation of the two Notch ligands. A similar decrease in Dll4 expression and increase in Jag1 expression was observed in primary lung EC isolated from mice heterozygous for endothelial-specific ERG deletion^21^ (*Erg*^*cEC-het*^) (Supplementary Fig. 1g,h). In support of these findings, ERG overexpression in HUVEC induced a significant upregulation of Dll4 expression and downregulation of Jag1 expression (Supplementary Fig. 1i). Moreover, ERG overexpression was able to normalize the expression of both Dll4 and Jag1 in siERG-treated HUVEC (Supplementary Fig. 1i), confirming ERG’s role in regulating transcription of these genes.

Comparative bioinformatic analysis of the Dll4 promoter revealed the presence of highly conserved ERG DNA binding motifs (Fig. 2e). Analysis of chromatin immunoprecipitation sequencing (ChIP-seq) data for markers of active promoters, namely histone marks H3K4me3 and H3K27Ac and RNA polymerase (RNA pol) II occupancy (from ENCODE (Encyclopedia of DNA Elements)^31^), showed that the location of these marks correlates with the position of the ERG binding motifs (Fig. 2e). ChIP-qPCR confirmed direct interaction of ERG with the Dll4 promoter (Fig. 2f, region R1); ERG enrichment was decreased in cells treated with ERG siRNA, supporting specificity (Fig. 2f). Finally, ERG overexpression resulted in transactivation of Dll4 promoter activity in EC (Fig. 2g), confirming that ERG drives Dll4 promoter activity in EC.

A similar analysis of the Jag1 locus showed multiple regions enriched with highly conserved ERG DNA binding motifs (Fig. 2h), which co-localize with H3K4me3 and/or H3K27Ac and low RNA pol II occupancy. ChIP-qPCR confirmed ERG interaction at three of these regions (Fig. 2i) and ERG siRNA indicated specificity of the enrichment. Inhibition of ERG expression by siRNA significantly increased Jag1 promoter activity in HUVEC compared with control (Fig. 2j), confirming that ERG acts as a repressor of Jag1 transcription. These data demonstrate that ERG directly controls the balance of expression between Dll4 and Jag1 in differentiated EC *in vitro*.

### ERG controls Notch signalling in maturing vessels *in vivo*

**Figure 3:**
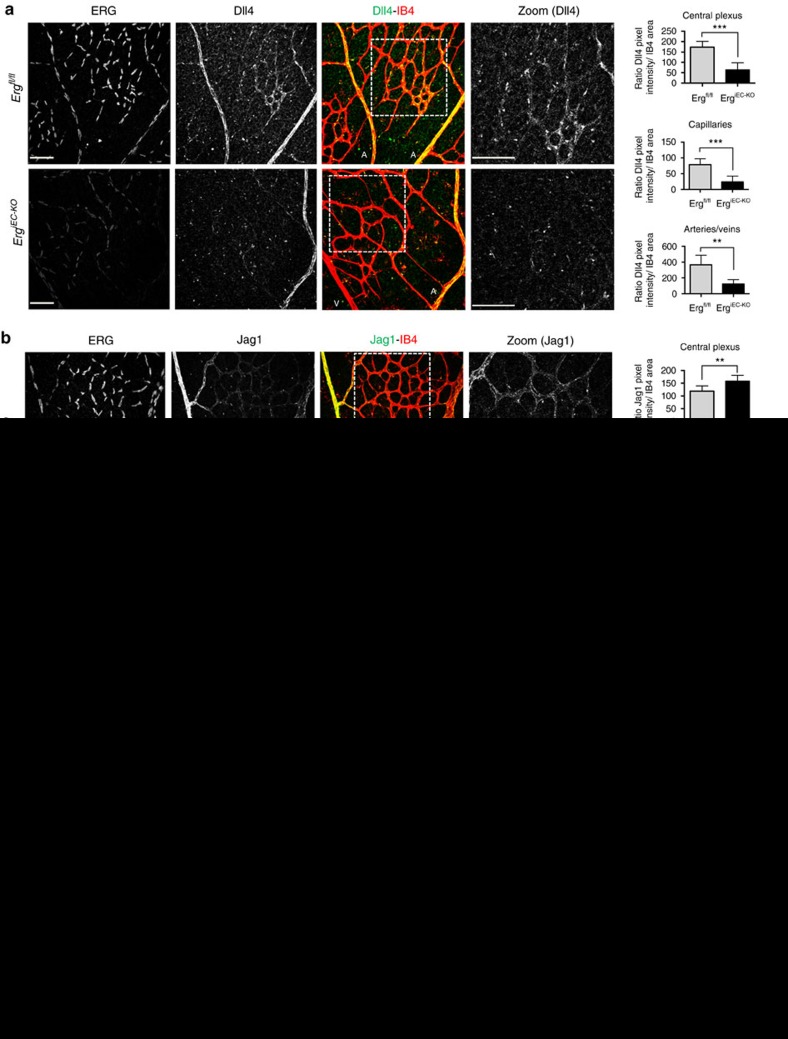
ERG controls Dll4 and Jag1 expression in the plexus of the mouse retina. Representative images and quantification of Dll4 and Jag1 (green) staining of postnatal day 6 retinal vessels in the (**a** and **b**) stable plexus and (**c** and **d**) angiogenic front from *Erg*^*fl/fl*^ and *Erg*^*iEC-KO*^ mice. Retinas are co-stained for isolectin B4 (IB4, red) and ERG (white, presented as a separate channel). Quantification represents the ratio between the sum of pixel intensity and isolectin B4 area (*n*=4 fields per mouse, *n*=4 mice per genotype). Scale bar, 70 μm. Arteries (A) and veins (V) are indicated. Arrowheads highlight tip cells at the angiogenic front. All graphical data are mean±s.e.m., *P<0.05, **P<0.01, ***P<0.001, Student's *t*-test.

We investigated whether ERG regulates Notch signalling during angiogenesis *in vivo*, using the retina postnatal neovascularization model in mice with inducible endothelial deletion of ERG (*Erg*^*iEC-KO*^). The phenotype of these mice has been recently reported^21^; effective downregulation of ERG expression was confirmed by immunofluorescence microscopy (Fig. 3a–d). In P6 retinas of *Erg*^*fl/fl*^ control mice, Dll4 and Jag1 were highly expressed in the arteries and to a lesser extent in the veins and capillaries of the central plexus (Fig. 3a,b and Supplementary Fig. 2a–d), while at the angiogenic front Dll4 was highly expressed by tip cells (Fig. 3c) and Jag1 was expressed by stalk cells (Fig. 3d), as previously reported^8^. Deletion of endothelial ERG resulted in decreased Dll4 expression (Fig. 3a, Supplementary Fig. 2a,c) and increased Jag1 expression (Fig. 3b, Supplementary Fig. 2b,d) in EC from the mature central plexus of the mouse retina, as measured globally (central plexus) and separately in capillaries and in the larger vessels (both arteries and veins). Surprisingly, expression of Dll4 and Jag1 at the angiogenic front was unaffected by ERG deletion (Fig. 3c,d). Consistent with this pattern of Notch ligand regulation, NICD expression was decreased in the central vascular plexus of *Erg*^*iEC-KO*^ mice compared to controls (Supplementary Fig. 3a), but not at the angiogenic front (Supplementary Fig. 3b). These data indicate that during angiogenesis, ERG regulates Notch signalling and the balance of expression between Dll4 and Jag1 selectively in the remodelling vascular plexus, where the process of vascular maturation and stabilization takes place.

Interestingly, ERG was found to regulate the balance of expression between Dll4 and Jag1 also in established vasculature. In retinas of 9-week-old *Erg*^*cEC-het*^ mice, when blood vessels have been remodelled to form a mature network, Dll4 expression was significantly downregulated (Supplementary Fig. 4a), whereas Jag1 expression was increased compared to control *Erg*^*fl/+*^ retinas (Supplementary Fig. 4b). These results indicate that ERG regulates Dll4 and Jag1 expression and Notch signalling selectively in the maturing vascular plexus and in established vasculature.

### ERG regulates Ang1-dependent Notch signalling *in vitro*

**Figure 4:**
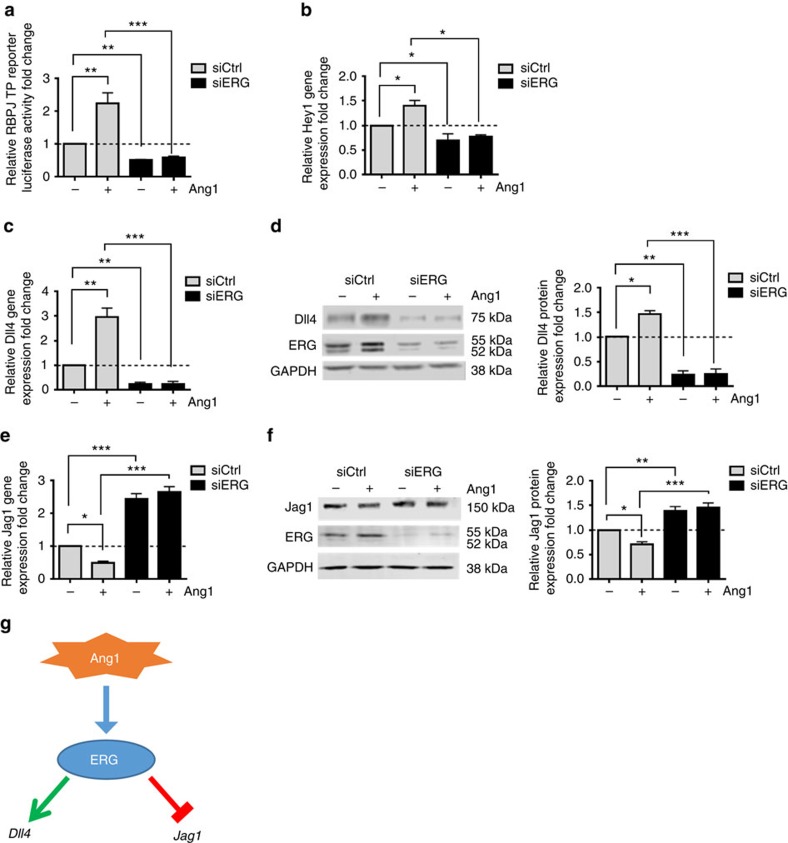
ERG is required for Ang1 regulation of Dll4 and Jag1 expression and Notch signalling. (**a**) Ang1-induced Notch transcriptional activity was determined by transfecting control and ERG-deficient cells with RBP-J TP1 reporter construct in the presence or absence of Ang1 (250 ng ml^−1^ for 6 h) (*n*=3). (**b**) Ang1-induced transcription of downstream Notch target gene Hey1 in control and ERG-deficient HUVEC (*n*=4). (**c**) qPCR and (**d**) WB analysis of Dll4 expression in extracts of siCtrl and siERG HUVEC treated in the presence or absence of Ang1 (250 ng ml^−1^ for 6 h) (*n*=5). (**e**) Jag1 mRNA and (**f**) protein expression in siCtrl and siERG HUVEC treated in the presence or absence of Ang1 (250 ng ml^−1^ for 6 h) (*n*=5). (**g**) Model: ERG mediates Ang1-dependent reciprocal regulation of Dll4 and Jag1 in endothelial cells. All graphical data are mean±s.e.m., *P<0.05, **P<0.01, ***P<0.001, Student's *t*-test.

A major pathway driving vascular maturation and stability is that of Ang1/Tie2 (ref. 32); in the developing retina, this pathway is active in the remodelling vascular plexus, where Tie2 is expressed, and not at the angiogenic front where EC do not express Tie2 (ref. 33). Ang1 has been shown to induce Dll4 expression and NICD signalling *in vitro*^18^,^34^; therefore, we investigated whether ERG mediates Ang1-dependent regulation of Notch signalling. In HUVEC, induction of Notch transcriptional activity by Ang1 treatment was inhibited by siRNA depletion of ERG, as shown by RBP-J luciferase reporter activity (Fig. 4a) and expression of the downstream target Hey1 (Fig. 4b). Depletion of ERG completely abolished Ang1-induced Dll4 gene (Fig. 4c) and protein expression (Fig. 4d). Interestingly, Ang1 treatment also decreased expression of Jag1 in an ERG-dependent manner, since the effect was lost in ERG-silenced HUVEC (Fig. 4e,f). These effects were not due to the inability of ERG-deficient cells to respond to Ang1, since Ang1-dependent Tie2 phosphorylation at cell–cell contacts and phosphorylation of Akt were similar in control and ERG-silenced HUVEC (Supplementary Fig. 5a,b), despite reduced levels of Tie2 in ERG-deficient cells (Supplementary Fig. 5b). Thus, ERG mediates Ang1-dependent activation of Notch and its reciprocal regulation of Dll4 and Jag1 in EC *in vitro* (Fig. 4g).

### ERG mediates Ang1-dependent blood vessel stability *in vivo*

**Figure 5:**
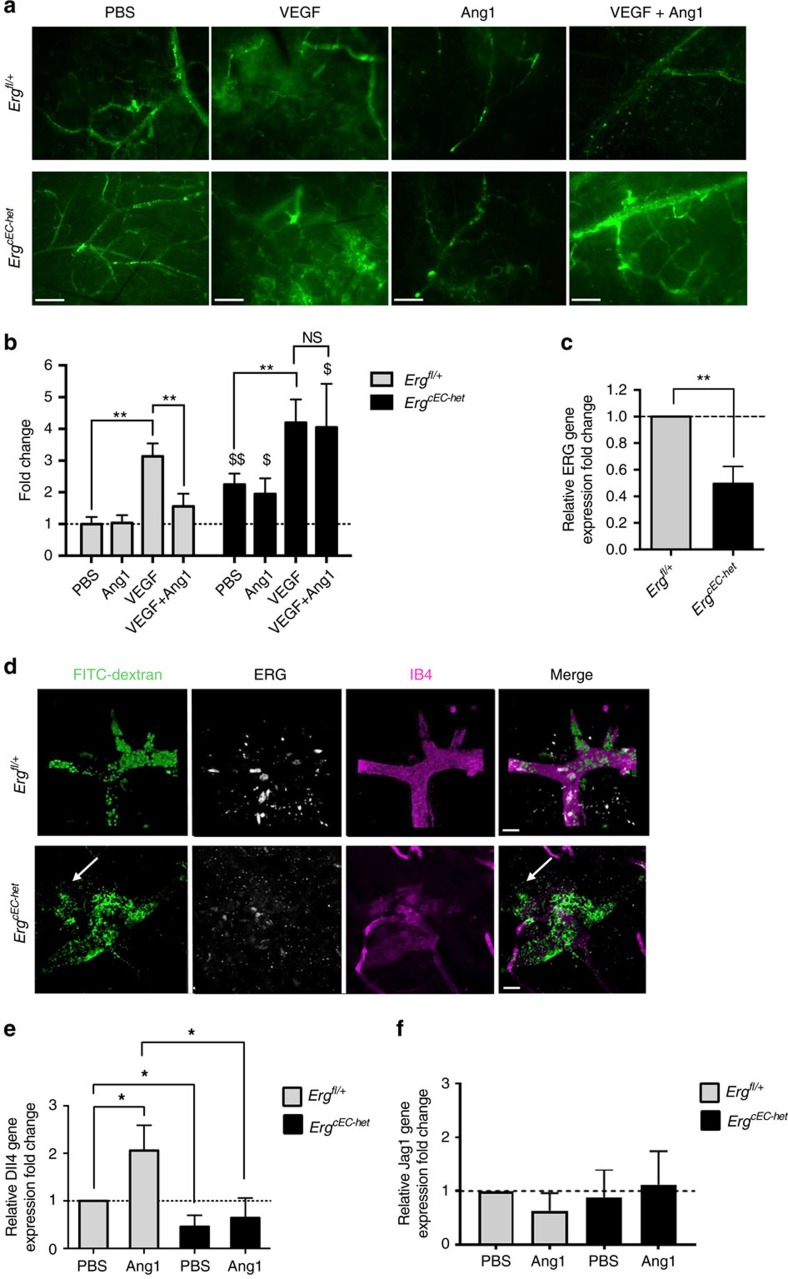
Ang1 requires ERG to promote vascular stability and drive Dll4 expression *in vivo.* (**a**) *Erg*^*fl/+*^ and *Erg*^*cEC-het*^ mice were subdermally injected with PBS, VEGF, Ang1, or a combination of reagents (Ang1+VEGF) (50 ng in 50 μl) for 1 h. Mice received intravenous injection of FITC-dextran 15 min before collecting skin samples. Representative images of whole-mount skin samples perfused with FITC-dextran (green). (**b**) Vessel permeability was quantified by measuring the intensity of FITC-dextran per field (*n*=5 fields per mouse, *n*=3 mice per genotype). The average of the mean intensity per mouse was converted to fold change compared to *Erg*^*fl/+*^ mice injected with PBS. (**c**) qPCR analysis of ERG expression in extracts of skin samples from control *Erg*^fl/+^ and ERG hemi-deficient (*Erg*^cEC-het^) mice (*n*=3). (**d**) Representative images of corresponding skin samples showing extravasation of FITC-dextran (green) from blood vessels stained for isolectin B4 (IB4, purple). qPCR analysis of (**e**) Dll4 and (**f**) Jag1 expression in extracts of skin samples from control *Erg*^*fl/+*^ and ERG hemi-deficient (*Erg*^*cEC-het*^) mice treated with intradermal injection of PBS or Ang1 for 1 h (*n*=3). All graphical data are mean±s.e.m., *P<0.05, **P<0.01, ***P<0.001, Student’s *t*-test. $: *Erg*^*cEC-het*^ mice compared to *Erg*^*fl/+*^ mice for each treatment, $ *P*<0.05, $$ *P*<0.01, $$$ *P*<0.001, Student’s *t*-test.

To establish whether ERG mediates Ang1-dependent responses *in vivo*, we used a VEGF-dependent permeability model, a well-established readout of the stabilizing effect of Ang1 on the vasculature^13^,^17^,^35^,^36^, in control (*Erg*^*fl/+*^) and *Erg* hemi-deficient (*Erg*^*cEC-het*^) mice. Heterozygous deletion of ERG was confirmed by qPCR (Fig. 5c) and immunofluorescence microscopy (Fig. 5d). Subcutaneous administration of VEGF in control *Erg*^*fl/+*^ mice caused increased dermal vascular permeability, which was prevented by co-injection with Ang1, as expected^13^,^17^,^34^,^35^ (Fig. 5a,b). Crucially, in *Erg*^*cEC-het*^ mice Ang1 was unable to reduce the VEGF-dependent increase in vascular permeability (Fig. 5a,b), demonstrating that ERG is required for Ang1 stabilizing activity *in vivo.* Interestingly, *Erg*^*cEC-het*^ mice also showed increased basal vascular permeability (Fig. 5b,d, and Supplementary Movies 1 and 2), in line with the reported role of ERG in controlling vascular permeability^21^,^37^.

We next examined whether ERG mediates Ang1-dependent regulation of Notch signalling *in vivo*. Dll4 and Jag1 mRNA levels were analysed in skin samples from *Erg*^*cEC-het*^ mice and littermate *Erg*^*fl/+*^ controls treated with Ang1 or PBS. Ang1 treatment resulted in upregulation of Dll4 expression in skin samples from *Erg*^*fl/+*^ mice; however, this was lost in *Erg*^*cEC-het*^ mice (Fig. 5e), in line with the *in vitro* data on ERG-depleted HUVEC. In contrast, despite a trend towards regulation, expression of Jag1 was neither significantly downregulated in *Erg*^*fll+*^ mice nor significantly upregulated in *Erg*^*cEC-het*^ mice after Ang1 treatment (Fig. 5f), possibly because Jag1 expression is not restricted to endothelial cells^38^. These results indicate that ERG is required for Ang1-dependent control of Dll4 expression *in vivo*.

### Ang1 increases ERG recruitment to Dll4 enhancers and promoter

**Figure 6:**
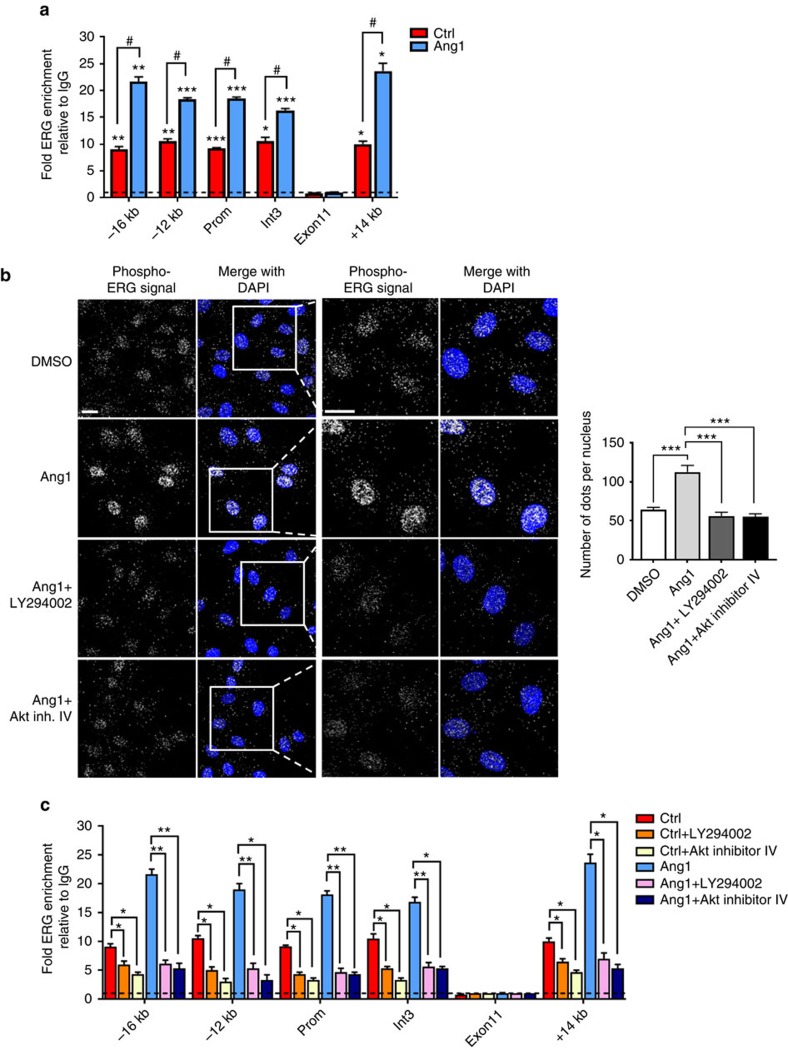
Ang1 induces ERG phosphorylation and binding to the Dll4 locus via PI3K/Akt. (**a**) ChIP-qPCR assays of confluent HUVEC treated with Ang1 (250 ng ml^−1^). ERG or IgG-immunoprecipitated DNA was analysed by qPCR with primers to −16 kb, −12 kb, promoter, intron 3 and +14 kb of Dll4 locus and negative control region exon 11. Results are expressed as fold change compared to IgG (*n*=3). *P<0.05, **P<0.01, ***P<0.001 indicates significant ERG enrichment compared to IgG and ^#^*P*<0.05 indicates significant change in Ang1-induced ERG enrichment compared to control conditions. (**b**) Confluent HUVEC were pre-treated with LY294002 (20 μM) or Akt inhibitor IV (8 μM) and treated with Ang1 (250 ng ml^−1^) or DMSO for 30 min. Proximity ligation assay analysis of ERG phosphorylation was performed using rabbit anti-ERG and mouse anti-phospho-serine antibodies (*n*>97 cells quantified per condition). Scale bar, 20 μm. (**c**) ChIP-qPCR analysis of HUVEC treated with Ang1 and LY294002 or Akt inhibitor IV. ERG or IgG-immunoprecipitated DNA was analysed by qPCR with primers to the Dll4 regulatory regions indicated and negative control region exon 11. Results are expressed as fold change compared to IgG (*n*=4). Graphical data are mean±s.e.m., *P<0.05, **P<0.01, ***P<0.001, Student's *t*-test.

Having established that ERG is essential for Ang1-dependent Dll4 expression in maturing and stable vasculature, we set out to investigate the molecular mechanisms for its regulation. As shown above, ERG binding to the Dll4 promoter is functionally active (see Fig. 2e–g). Four putative enhancer regions within the Dll4 locus were identified by Sacilotto *et al*.^39^; these are located at −16 and −12 kb upstream of the transcription start site (TSS), within the third intron of Dll4 and 14 kb downstream of the TSS (denoted as −16, −12, int3 and +14)^39^. Comparative bioinformatic analysis combined with ENCODE data identified conserved ERG DNA binding motifs at all four putative enhancer regions enriched in H3K27Ac and H3K4me1 histone modifications and DNAse I hypersensitivity (Supplementary Fig. 6). ERG binding to the −16, −12, int3 and +14 kb regulatory regions within the Dll4 locus was confirmed by ChIP-qPCR (Fig. 6a); Ang1 increased ERG binding to the Dll4 promoter and enhancers by approximately twofold (Fig. 6a). Thus ERG regulates expression of Dll4 through binding to the promoter and multiple enhancer regions, which is enhanced by Ang1.

### Ang1 induces ERG phosphorylation via the PI3K/Akt axis

We have shown that Ang1 increases ERG binding to the Dll4 locus. This is unlikely to be mediated solely by increased ERG expression, since Ang1 had a modest effect on ERG levels in HUVEC *in vitro* and *in vivo* (Supplementary Fig. 7a–c).

Ang1 stimulation of confluent endothelial monolayers has been shown to result in preferential activation of the PI3K/Akt pathway^34^. Also, Ang1-dependent upregulation of Dll4 is mediated by PI3K/Akt signalling^18^. ERG has been shown to be phosphorylated at serine residues, in non-EC^40^,^41^. Therefore, we investigated whether Ang1 could induce ERG phosphorylation in EC through the PI3K/Akt pathway. Ang1 treatment in HUVEC induced ERG phosphorylation at serine residues, as shown by proximity ligation assay (PLA)^42^; Ang1-dependent ERG phosphorylation was localized within the EC nucleus (Fig. 6b and Supplementary Fig. 7d). Interestingly, treatment with either PI3K inhibitor LY294002 or Akt inhibitor IV completely abolished Ang1-induced ERG phosphorylation (Fig. 6b). These data indicate that Ang1 is able to induce ERG phosphorylation via the PI3K/Akt axis.

Next, we examined whether the PI3K/Akt pathway mediates binding of ERG to the regulatory regions in the Dll4 gene locus in response to Ang1. ChIP-qPCR analysis was performed on confluent Ang1-treated EC, in the presence of LY294002 or Akt inhibitor IV. Ang1-induced ERG enrichment at these loci was ablated by PI3K or Akt inhibitors (Fig. 6c). These results identify a novel Ang1-PI3K/Akt-ERG signalling axis in the control of Dll4 expression in confluent EC.

Interestingly, baseline ERG binding to the Dll4 locus and transactivation of Dll4 promoter activity were also dependent on PI3K/Akt (Fig. 6c and Supplementary Fig. 7e); PLA analysis also showed basal phosphorylation of ERG at serine residues (Fig. 6b). These results suggest the presence of a constitutive PI3K/Akt-ERG-dependent pathway in confluent EC *in vitro*, which is potentiated by Ang1.

### An ERG-β-catenin-NICD complex controls Dll4 expression

**Figure 7:**
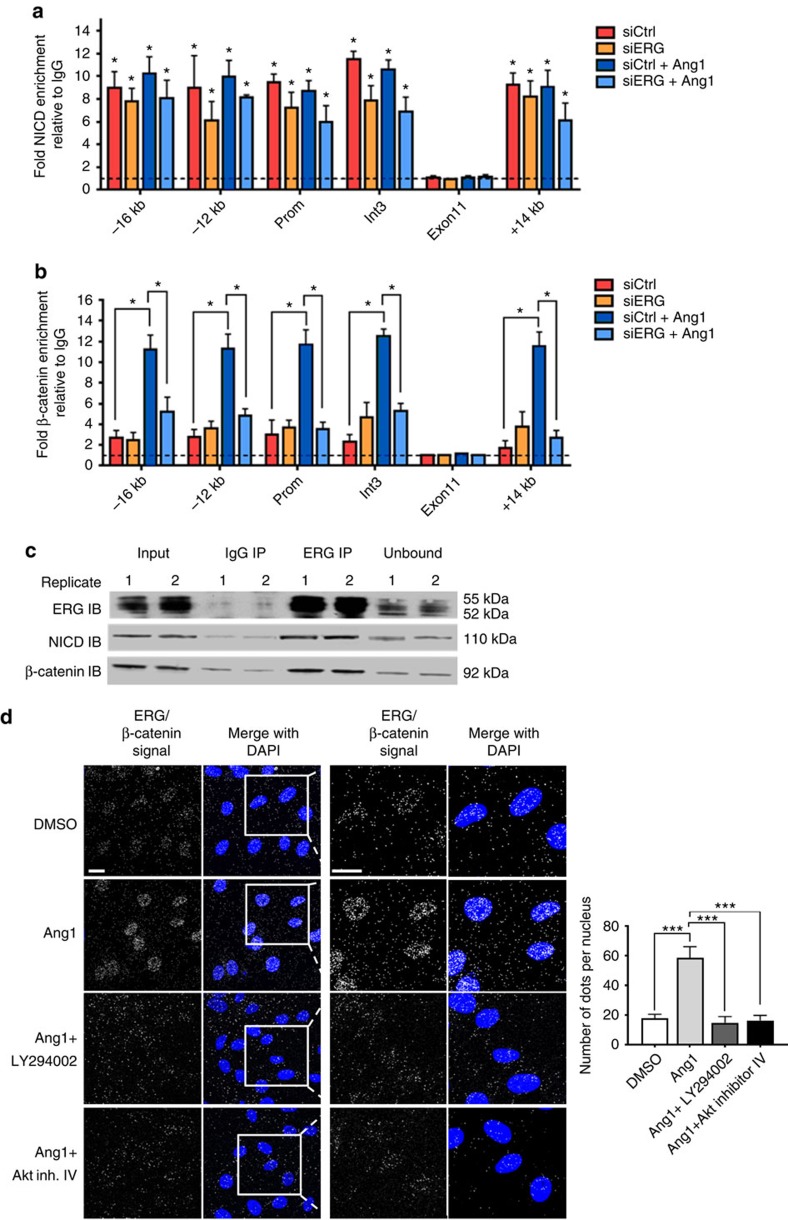
Ang1 induction of β-catenin occupancy at Dll4 promoter and enhancers requires ERG. ChIP-qPCR analysis of siCtrl and siERG-treated HUVEC, in the presence or absence of Ang1 (250 ng ml^−1^). Chromatin was immunoprecipitated with (**a**) an anti-NICD antibody, (**b**) an anti-β-catenin antibody or control IgG. Immunoprecipitated DNA was analysed by qPCR with primers to −16 kb, −12 kb, promoter, intron 3 and +14 kb of Dll4 locus. Primers covering a negative control region within exon 11 were also used. Results are expressed as fold change enrichment compared to IgG (*n*=3). (**c**) HUVEC lysates were immunoprecipitated with an anti-ERG antibody. Immunoprecipitates were analysed by immunoblotting (IB) with anti-ERG, anti-NICD and anti-β-catenin antibodies. (**d**) Confluent HUVEC were pre-treated with LY294002 (20 μM) or Akt inhibitor IV (8 μM) and treated with Ang1 (250 ng ml^−1^) or DMSO for 30 min. Proximity ligation assay analysis of localization of ERG-β-catenin interaction was performed using rabbit anti-ERG and mouse anti-β-catenin antibodies (*n*>99 cells quantified per condition). Scale bar, 20 μm. All graphical data are mean±s.e.m., *P<0.05, **P<0.01, ***P<0.001, Student's *t*-test.

The data described so far point towards a role for ERG in controlling Ang1-dependent activation of the Notch pathway in the maturing vasculature. We have recently shown that ERG is required for vascular maturation and stability by controlling Wnt/β-catenin signalling^21^. Crosstalk between Notch and Wnt signalling has been reported in multiple systems^9^,^43^,^44^,^45^; moreover, Zhang *et al*. have shown that Ang1 promotes Dll4 expression through Akt-mediated activation of β-catenin *in vitro*^18^. Thus we speculated that ERG may act to coordinate these pathways in EC at the transcriptional level. We investigated this hypothesis using ChIP-qPCR in confluent EC, in the presence or absence of Ang1. NICD enrichment at the Dll4 promoter and enhancer regions was detected in baseline conditions and was unaffected by inhibition of ERG expression or by Ang1 treatment (Fig. 7a). In contrast, basal β-catenin enrichment at the Dll4 promoter and enhancers was low and was increased by Ang1 (Fig. 7b). Crucially, Ang1 dependent β-catenin enrichment requires ERG, since it was abolished in siERG-treated HUVEC (Fig. 7b). These findings indicate that ERG is required for Ang1-dependent β-catenin activation and recruitment of β-catenin at the Dll4 locus.

We have previously shown that β-catenin levels are decreased in ERG-deficient cells, due to decreased protein stability^21^. To test whether the reduced recruitment of β-catenin to the Dll4 promoter in ERG-deficient cells was simply due to reduced levels of β-catenin, we stabilized β-catenin protein levels with lithium chloride (LiCl), as before^21^. LiCl normalized β-catenin protein levels in ERG-deficient cells (Supplementary Fig. 8a); however, it was unable to normalize expression of Dll4 (Supplementary Fig. 8b). These results confirm that ERG is required for β-catenin to control Dll4 expression.

β-catenin has been previously shown to form a complex with NICD/RBP-J on the Dll4 intron3 enhancer^18^, where ERG also binds. Thus we examined the possibility that ERG may be part of the complex with β-catenin and NICD. Co-immunoprecipitation from confluent HUVEC extracts showed that ERG associates with both endogenous β-catenin and NICD (Fig. 7c). PLA analysis in unstimulated confluent HUVEC confirmed the interaction between ERG and β-catenin, and localized it in the nucleus (Fig. 7d, top). Interestingly, Ang1 treatment significantly increased the interaction between ERG and β-catenin (Fig. 7d); the ERG-β-catenin complex was inhibited by treatment with either PI3K or Akt inhibitors (Fig. 7d). Thus ERG can form a complex with β-catenin and NICD, which is enhanced by Ang1 via the PI3K/Akt pathway. These data indicate that this complex is required to drive Dll4 expression and Notch signalling in confluent EC.

### A positive Notch-ERG loop regulates Dll4 expression

**Figure 8:**
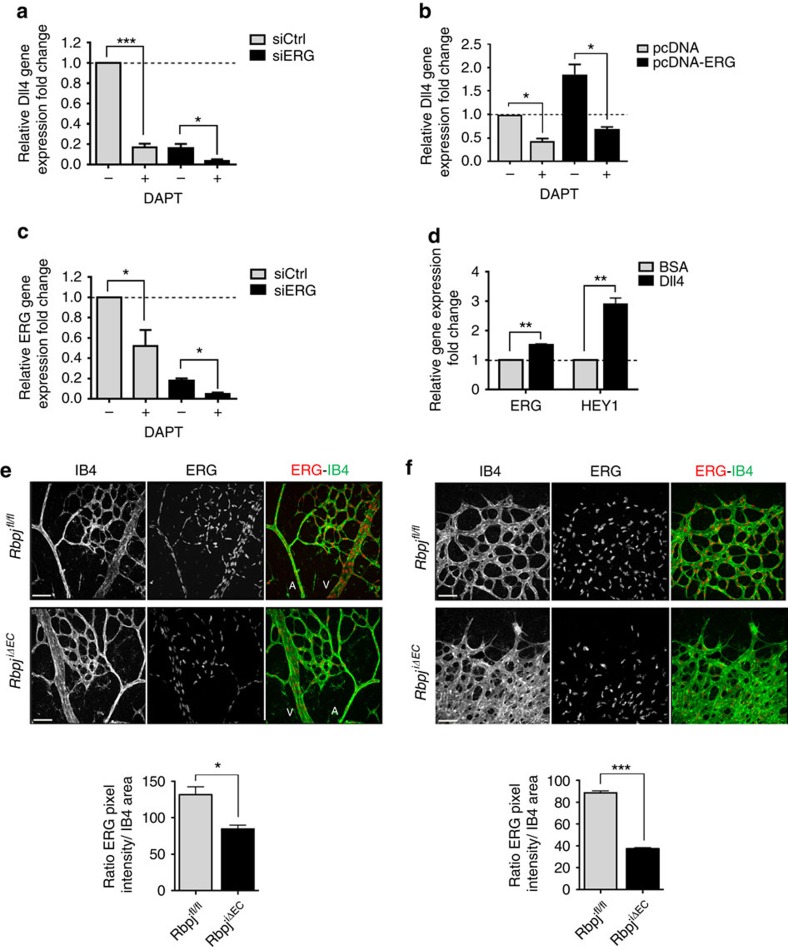
Reciprocal regulation of Notch signalling and ERG expression. (**a**) Dll4 mRNA expression in siCtrl and siERG-transfected HUVEC treated in the presence or absence of the γ-secretase inhibitor DAPT (*n*=4). (**b**) Dll4 mRNA expression in HUVEC transfected with ERG cDNA expression plasmid (pcDNA-ERG) or an empty expression plasmid (pcDNA) and treated in the presence or absence of the γ-secretase inhibitor DAPT (*n*=3). (**c**) ERG mRNA expression in siCtrl and siERG-transfected HUVEC treated in the presence or absence of DAPT (*n*=4). (**d**) mRNA expression of ERG and the Notch target gene Hey1 in HUVEC stimulated with control BSA or Dll4 (*n*=4). Representative images and quantification of ERG (red) staining of P6 retinal vessels in the (**e**) vascular plexus and (**f**) angiogenic front from control (*Rbpj*^*fl/fl*^) and *Rbpj*^*i*Δ*EC*^ mice. Retinas are co-stained for isolectin B4 (IB4, green). Quantification represents the ratio between the sum of pixel intensity and isolectin B4 area (*n*=4 fields per mouse, *n*=4 mice per genotype). Scale bar, 70 μm. Arteries (A) and veins (V) are indicated. All graphical data are mean±s.e.m., *P<0.05, **P<0.01, ***P<0.001, Student's *t*-test.

Notch signalling itself is required for Dll4 regulation^18^,^46^; therefore, we tested whether ERG and NICD cooperate to regulate Dll4. In HUVEC, depletion of NICD by DAPT, a γ-secretase inhibitor, caused a profound decrease in Dll4 expression (Fig. 8a), as expected^18^. Interestingly, this was similar to what was observed with ERG depletion and combination of DAPT and ERG siRNA caused a further significant decrease in Dll4 expression (Fig. 8a). In line with this observation, the increased Dll4 mRNA caused by ERG overexpression was significantly reduced by DAPT (Fig. 8b and Supplementary Fig. 9). These results indicate that ERG and Notch signalling are jointly required for Dll4 expression.

Finally, DAPT treatment of HUVEC decreased ERG transcript levels by 50% compared to control (Fig. 8c), while Dll4 stimulation of HUVEC significantly upregulated ERG mRNA levels, as well as those of the Notch target gene Hey1, as expected (Fig. 8d). These data suggest that Notch signalling regulates ERG expression. To confirm these findings *in vivo*, we investigated ERG expression in the retinal vasculature of an inducible EC-specific knockout model of the *Rbpj* gene (*Rbpj*^*i*Δ*EC*^)^47^,^48^, which encodes the transcription factor RBP-Jκ, the effector of Notch-induced gene expression^49^. Inactivation of *Rbpj* led to a downregulation of ERG protein expression *in vivo*, both in the retinal central vascular plexus (Fig. 8e) and at the angiogenic front (Fig. 8f). These results identify a positive feedback loop between Notch and ERG signalling, both essential pathways for vascular maturation and homeostasis.

**Figure 9:**
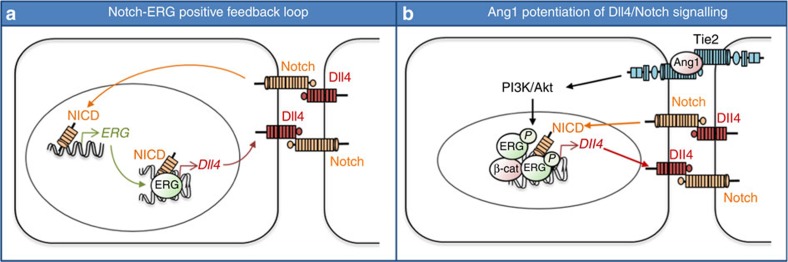
Model: ERG mediates Ang1 potentiation of Dll4/Notch signalling. (**a**) Notch-ERG positive feedback loop. ERG drives expression of the Notch ligand Dll4 and is required for endothelial Notch signalling. Notch signalling itself upregulates ERG expression, suggesting that continued Dll4 expression and Notch signalling is maintained through this positive feedback loop. (**b**) ERG is required for Ang1 induction of Dll4. In confluent cells, Ang1/Tie2 signalling induces PI3K/Akt-dependent ERG phosphorylation (P). This increases ERG binding to the Dll4 gene locus and recruitment of β-catenin. The complex of ERG with NICD and β-catenin mediates Ang1-dependent Dll4/ Notch signalling in confluent EC.

In summary, the data presented here suggest a new model for the control of Dll4/Notch signalling in the endothelium, selectively within maturing and established vessels (Fig. 9). Here, ERG and NICD form a complex which controls endothelial Dll4/Notch signalling to promote maturation and stability. Notch itself upregulates ERG levels, thus promoting a positive ERG-Notch loop to sustain this pathway (Fig. 9a). Ang1 activation in adjacent EC results in activation of the PI3K/Akt pathway, which phosphorylates ERG, enhancing its binding to the Dll4 locus and promoting the recruitment of β-catenin (Fig. 9b). This transcriptional complex is required to drive Dll4 expression and ultimately enhance Notch signals, promoting vascular maturation and stability.

## Discussion

In this study, we identify ERG as an endothelial transcriptional effector activated downstream of Ang1 to promote Notch signalling and vascular stability. We show that ERG is required for Notch signalling *in vitro* and *in vivo*. ERG controls the balance of expression between the Notch ligands Dll4 and Jag1 selectively in the remodelling vascular plexus, which is exposed to Ang1/Tie2 signals, and not at the angiogenic front, where Tie2 levels are low^33^. We show that Ang1 is able to phosphorylate ERG via PI3K/Akt, and that this promotes the formation of an ERG-β catenin complex, which binds multiple sites in the Dll4 locus and drives Dll4 expression. Finally, we identify an ERG-Notch positive loop, which sustains the pathway.

The two Notch ligands Dll4 and Jag1 display distinct spatial expression patterns and play opposing functional roles in angiogenesis^8^. In this study, we show that ERG drives Dll4 while repressing Jag1 expression. To our knowledge, this is the first time that reciprocal transcriptional control of Notch proteins by a single transcription factor has been shown in EC. Interestingly, the phenotype of ERG loss *in vivo* does not phenocopy either endothelial loss of Dll4 or gain of Jag1 (refs 8, 30); indeed the overall outcome of the loss of Dll4/Jag1 balance appears to be defective angiogenesis. These different phenotypic outcomes may be due to the added effect of the loss of β-catenin activity, which is significantly reduced in ERG-deficient mice^21^; they could also be partly due to the dysregulation of multiple Notch-related proteins, such as Notch receptors or Fringe proteins. Interestingly, Fringe glycosyltransferases enhance the activation of Notch in response to Delta-like ligands, but have the opposite effect for Jagged ligands^8^,^50^. Boareto *et al*. recently used theoretical modelling to investigate the dynamic relationship between Dll4/Notch/Jag1 and proposed that conditions with Jag1 overexpression, Dll4 repression or Fringe inhibition (all observed in ERG-deficient cells) should lead to a hybrid tip/stalk phenotype resulting in pathological angiogenesis^51^.

Four putative enhancers for the Dll4 gene have been previously identified in HUVEC^39^. Here we demonstrate that these enhancer regions are all bound by ERG, alongside an ERG-enriched promoter region. Enhancer–promoter regulation is a fundamental mechanism underlying differential transcriptional regulation^52^,^53^,^54^. The involvement of ERG at multiple sites suggests a complex 3D structure and looping of the transcriptional unit regulating lineage-specific expression of Dll4. This could allow other factors to converge in the same genomic region, leading to changes in epigenetic marks and alterations in chromatin structure. Future experiments on the chromatin landscape and the dynamic binding of potential co-activators or co-repressors will explore this hypothesis.

It has been previously shown that Dll4 expression is maintained through a Notch-dependent positive feedback loop^46^. Here we find that Notch signalling regulates ERG expression, thus promoting a positive ERG-Notch loop to sustain this pathway. Notch regulates multiple steps of vascular development; it has been suggested that Notch signalling acquires specificity through the formation of transcriptional complexes with other factors^39^. Previous work has shown that the Ang1-PI3K/Akt axis induces the formation of a transcriptional complex between β-catenin/NICD/RBP-J to enhance Dll4 expression and Notch signalling^18^,^45^. Notch and β-catenin are also expressed in nonvascular tissues, while ERG’s expression profile is restricted to the endothelium and a very limited number of other lineages. Thus ERG may confer endothelial-specificity to this pathway by mediating the Ang1/Tie2 signals to induce the formation of a β-catenin-NICD complex, which cooperatively induces Dll4 expression in the maturing and established endothelium. Given ERG’s multiple roles in angiogenesis and homeostasis, it is likely that ERG itself engages in cooperation with distinct transcriptional complexes depending on different extracellular signals, to achieve specific outcomes. Crucially, our data show that Ang1 regulates endothelial ERG function via the PI3K/Akt pathway. Despite its key role in multiple endothelial functions, very little is known about the regulation of ERG function; future studies manipulating ERG to interfere with its activation state will provide important support to the currently available endothelial deletion approaches.

Previous work has implicated ERG in the VEGF-dependent regulation of Dll4 expression in developing arteries^55^. Arterial-venous patterning in the retinas from P6 *Erg*^*iEC-KO*^ mice appears morphologically normal, with only rarely observed arteriovenous shunts typically associated with disrupted arterial specification. These observations are consistent with those described by Wythe *et al*.^55^, who reported no gross morphological defects in the arteries or veins of *Erg* endothelial-isoform-specific knockout mice. Further detailed molecular characterization will be needed to clarify the role of ERG in arterial-venous differentiation.

During angiogenesis, VEGF is a key driver of Dll4 expression in tip cells; however, in our study Dll4 expression in ERG-deficient retinas was unaffected at the angiogenic front. This is in line with a recent study by the De Val group^56^, showing that ETS motifs within the Dll4 intron 3 enhancer are not required for its expression at the angiogenic front. The authors suggest a model in which MEF2 transcription factors cooperate with ETS factors, where ETS provide essential endothelial expression information and MEF2 contribute angiogenic sprout specificity^56^. Ang1 can also promote Dll4 expression, and here we show that this requires ERG. Tie2 expression pattern studies suggest that the Ang1/Tie2 pathway is active in the remodelling, maturing plexus, and indeed loss of endothelial ERG results in decreased Dll4/Notch signalling in the remodelling plexus. These studies suggest a model of context-specific combinatorial networks which integrate growth factor signals to assemble distinct transcriptional complexes in different phases of vascular development and angiogenesis.

## Methods

### Mice and breeding

The ERG conditional knockout mouse models were generated as described previously^21^. *Erg*^*fl/fl*^ mice were crossed with the following endothelial-specific Cre transgenic deleter lines: *Cdh5(PAC)-*CreERT2 (ref. 57) and *Tie2*-Cre (ref. 58). For *Rbpj* targeting in the endothelium, *Rbpj*^*flox*^ mice^49^ were combined with EC-specific *Pdgfb-iCre* transgenic deleter line^59^. All animal experiments were conducted according to Imperial College London-approved protocols, in compliance with the UK Animals (Scientific Procedures) Act of 1986. All animals used were maintained on a C57BL/6 background. Both male and female mice were used for experiments and were 6 days or 9–10 weeks old. All experiments were conducted using littermate controls.

### *In vivo* permeability assay

*In vivo* permeability assay was performed as previously described^36^. Briefly, PBS, VEGF, Ang1 (kindly provided by Regeneron Pharmaceuticals, Inc.) or VEGF and Ang1 were injected intradermally (50 ng in 50 μl) for 1 h in four distinct regions of the abdomen of 10-week-old *Erg*^*fl/+*^ and *Erg*^*cEC-het*^ mice. Fifteen minutes before the killing, the mice received an intravenous administration of high molecular weight FITC-Dextran (2 × 10^6^ MW). Skin samples were dissected and fixed with paraformaldehyde (4%) for 1 h. Epidermis was removed from the skin and samples were imaged whole-mount. Mean fluorescence intensity for FITC-Dextran was quantified in five fields per mouse. An average of the mean intensity per mouse was converted to fold change compared to *Erg*^*fl/+*^ mice injected with PBS. Some skin samples were processed for immunofluorescence staining or digested with proteinase K (Qiagen) for 45 min at 56 °C and used for RNA extraction.

### Immunofluorescence analysis of mouse tissue

Mice were injected intraperitoneally (i.p.) with tamoxifen (50 μg per mouse; Sigma) at postnatal (P) day 1, P2 and P3. Retinas were collected from P6 *Erg*^*fl/fl*^ and *Erg*^*iEC-KO*^ mice and from 9-week-old *Erg*^*fl/+*^ and *Erg*^*cEC-het*^ mice and processed for immunofluorescence staining as described previously, with minor modifications^60^. Briefly, whole eyes were fixed with freshly prepared 4% paraformaldehyde/PBS for 1 h 45 min on ice. Retinas were dissected and blocked in retina-blocking buffer (1% bovine serum albumin (BSA), 0.3% Triton, PBS) overnight at 4 °C. Retinas were washed three times for 10 min with Pblec buffer (1 mM CaCl_2_, 1 mM MgCl_2_, 1 mM MnCl_2_, 1% Triton X-100 in PBS), and then incubated overnight at 4 °C with biotinylated Griffonia simplicifolia lectin I (isolectin B4) (B1205, 1:250, Vector Laboratories) and the following primary antibodies: rabbit anti-ERG (ab110639, 1:200, Abcam), goat anti-Dll4 (AF1389, 1:50, R&D systems), goat anti-Jag1 (J4127, 1:50, Sigma) and rabbit anti-cleaved Notch1 (Val 1744, #2421, 1:100, Cell Signalling) (anti-NICD). The following day, retinas were washed twice for 15 min in washing buffer (1:1 retina-blocking buffer/PBS) and twice (15 min per wash) in PBS, then incubated at room temperature for 2 h with Alexa Fluor streptavidin conjugates or species-specific Alexa Fluor-coupled secondary antibodies (all at 1:500, Invitrogen) diluted in blocking buffer. For NICD immunostaining, biotinylated goat anti-rabbit IgG (1:100, Vector Laboratories) was used followed by TSA-Cy3 (Perkin Elmer). Retinas were washed twice for 15 min in washing buffer and twice for 10 min in PBS, re-fixed in 4% paraformaldehyde for 15 min at room temperature and washed once in PBS/0.1% Triton X-100 and then in PBS, before they were flat-mounted on glass microscope slides using Fluoromount-G (Southern Biotech). Confocal microscopy was carried out on Leica TCS SP5 and Carl Zeiss LSM780 confocal microscopes.

### Mouse retina analysis and quantification

Retinal vasculature Z-stack images were analysed with Volocity software (PerkinElmer). For Dll4 or Jag1 staining, quantification of the pixel intensity (arbitrary units) was performed using the ‘Find Objects’ tool of Volocity. Evaluation of vascular NICD staining was performed by quantifying only the signal (pixel intensity, arbitrary units) overlapping IB4-positive vascular structures using the ‘Find Objects’ tool. Each pixel intensity value was normalized to isolectin B4 area (μm^2^). The data are presented as the ratio between the pixel intensity and isolectin B4 area.

### Cell culture

Primary HUVEC were collected from umbilical cords by standard procedures in strict accordance with established guidelines and cultured in supplemented M199 media, as previously described^21^. Human ERG expression was inhibited using 20 nM siRNA against ERG exon 6 (Qiagen; 5′-CAGATCCTACGCTATGGAGTA-3′) or a second siRNA (#2) targeting exon 7 (Invitrogen; 5′-ACTCTCCACGGTTAATGCATGCTAG-3′) of the ERG locus, both denoted as siERG in the text. In parallel, an AllStars Negative Control siRNA (Qiagen) was used, which are denoted as siCtrl. For rescue experiments, HUVEC were transfected with control or ERG siRNA (20 nM). After 6 h, the media was replaced and HUVEC were transfected with pcDNA3.1 empty vector (used as a control) or pcDNA-ERG expression plasmid using Viromer Yellow Transfection Reagent (Lipocalix). Cells were collected after 18 h and RNA extracted (Qiagen).

### Pharmacological/growth factor *in vitro* cell treatments

HUVEC were serum starved in M199 containing 1% BSA for 6 h and were stimulated with 250 ng ml^−1^ human modified Ang1 (kindly provided by Regeneron Pharmaceuticals, Inc.) unless specified in the figure legends. In some experiments, cells were pre-treated in the presence of 20 μM LY294002 (Cell Signaling Technology), or 8 μM Akt inhibitor IV (Calbiochem) for 30 min.

### Dll4 stimulation of endothelial cells

Lyophilized recombinant human Dll4 (R&D Systems) was reconstituted at 100 μg ml^−1^ in PBS containing 0.1% BSA. For stimulation of cultured endothelial cells, Dll4 was immobilized by coating culture dishes with 500 ng ml^−1^ Dll4 in PBS for 1 h at room temperature or overnight at 4 °C.

### Isolation of mouse lung endothelial cells

Primary mouse endothelial cells were isolated from the lungs of control *Erg*^*fl/+*^ and *Erg*^*cEC-het*^ mice. Lungs were minced using GentleMACS C tubes and GentleMACS Dissociator (Miltenyi Biotec), digested with 0.1% collagenase type I (Invitrogen, UK), and sieved through a 70 μm-pore cell strainer (BD Falcon). EC were selected by magnetic immunosorting (Dynabeads; Invitrogen) with a negative sort for FcγRII/III receptor-positive macrophages and a positive sort for ICAM-2-positive endothelial cells. Cells were cultured in EGM-2 media (Lonza), in flasks pre-coated with a mixture of 0.1% gelatin (Sigma), PureCol (Invitrogen) and human plasma fibronectin (Chemicon).

### Real-time polymerase chain reaction

RNA extraction from mouse tissues, primary lung EC and HUVEC was carried out using the RNeasy kit (Qiagen). First strand cDNA synthesis was carried out using Superscript III Reverse Transcriptase (Invitrogen). Quantitative real-time PCR was performed using PerfeCTa SYBR Green Fastmix (Quanta Biosciences) on a Bio-Rad CFX96 system. Gene expression values were normalized to GAPDH expression (human) or HPRT (mouse). See Supplementary Table 1 for list of oligonucleotides used in this study.

### Immunoblotting analysis

Whole cell protein lysates were prepared from HUVEC using CelLytic reagent (Sigma). Immunoblotting of cell lysates was performed according to standard conditions. Immunoblots were labelled with the following primary antibodies: anti-Akt (11E7) (4685, 1:1,000, Cell Signaling Technology), anti-phospho (S473)-Akt (9271, 1:1,000, Cell Signaling Technology), anti-ERG (sc353, 1:500, Santa Cruz Biotechnology), anti-ERG (ab133264, 1:1,000, Abcam), anti-Dll4 (1:500, R&D systems), anti-GAPDH (MAB374, 1:10,000, Millipore), anti-Jag1 (sc-6011, 1:1,000, Santa Cruz Biotechnology), anti-NICD/cleaved Notch1 (Val1744) (2421, 1:500, Cell Signaling), anti-Tie2 (D9D10) (7473, 1:1,000, Cell Signaling). Primary antibodies were detected using fluorescently labelled secondary antibodies: goat anti-rabbit IgG DyLight 680 and goat anti-mouse IgG Dylight 800 (Thermo Scientific). Detection and quantification of fluorescence intensity were performed using an Odyssey CLx imaging system (LI-COR Biosciences, Lincoln) and Odyssey 2.1 software. In some instances, HRP-conjugated secondary antibodies were used for chemiluminescence detection and protein levels were quantified by densitometry and normalized against loading controls. See Supplementary Fig. 10 for the uncropped immunoblots.

### Immunoprecipitations

Confluent HUVEC were collected in RIPA lysis buffer (20 mM Tris-HCl pH 7.5, 150 mM NaCl and 0.5% Triton X-100) supplemented with Phenylmethanesulfonyl Fluoride and Protease inhibitor cocktail (Sigma). Either 2 μg ERG rabbit polyclonal antibody (H-95; sc-28680, Santa Cruz Biotechnology) or negative control rabbit IgG in buffer (#7074; Millipore, UK) was incubated with protein A sepharose beads (Sigma, UK) on an end-to-end rotator for 2 h at 4 °C. The antibody-protein A sepharose complexes were then incubated with pre-cleared cellular lysates (800 μg) for at least 2.5 h or overnight at 4 °C. The immuno-complexes were detected by western blot with goat anti-ERG (1:500, Santa Cruz), mouse anti-β-catenin (clone 17, 610153, 1:200, BD Bioscience) and rabbit anti-NICD (Cleaved Notch1 (Val1744) Antibody, 2421, Cell Signaling) antibodies. Primary antibodies were detected using HRP-conjugated secondary antibodies and chemiluminescence detection or using fluorescently labelled secondary antibodies. Fluorescence intensity detection was performed using an Odyssey CLx imaging system.

### Immunofluorescence analysis of HUVEC

HUVEC for immunofluorescence and PLA were fixed with 4% paraformaldehyde for 15 min and permeabilized for 3 min with 0.5% Triton X-100 in PBS before blocking with 3% BSA for 1 h. For immunofluorescence, cells were incubated with the following primary antibodies: mouse anti-ERG (sc-376293, 1:200, Santa Cruz) and rabbit anti-phospho-Tie2 (Y992) (AF2720, 1:500, R&D systems). Secondary antibodies used were anti-mouse AF 488 and anti-rabbit Texas Red (all from Invitrogen). Nuclei were visualized using DAPI. PLA was performed according to the manufacturer’s instructions using the Duolink *In Situ* Orange Kit Mouse/Rabbit (Sigma) and the following primary antibodies: rabbit-anti ERG (ab133264, 1:500, Abcam), mouse anti-β-catenin (clone 17, 610153, BD) and mouse anti-phosphoserine antibody (clone PSR-45, P5747, Sigma). Nuclei were visualized using DAPI. Confocal microscopy was carried out on a Carl Zeiss LSM780. Images were analysed with ImageJ (NIH) and Volocity (Version 6.3, PerkinElmer).

### Plasmids

A 1 kb region, including region R1 used for ChIP-qPCR, of the Dll4 promoter proximal to the transcription initiation site was PCR amplified from human genomic DNA and cloned into the pGL4.10[luc2] Luciferase Reporter Vector (Promega); see Supplementary Table 1 for oligonucleotide sequences. The human Jag1 promoter (−3,736 to +58 bp relative to TSS; including regions R1 and R2 used for ChIP-qPCR) was kindly provided by Christopher Hughes (University of California) and cloned into a pGL3 Luciferase Reporter Vector. Human ERG cDNA (NCBI Accession NM_182918) was cloned into the mammalian expression vector pcDNA3.1 (Invitrogen). The RBPJ TP-1 luciferase Notch-reporter was from U. Zimber-Strobl, Helmholtz Zentrum München. pGL4.10[luc2] (Promega, Madison, USA) Firefly Luciferase empty vector, lacking a promoter sequence, was used as a control. pGL4.73[hRluc/SV40] (Promega) *Renilla* luciferase vector was used as an internal control in the luciferase assay.

### Reporter assays

For siRNA experiments, cells were transfected with 20 nM ERG or control siRNAs for 24 h, followed by transfection with luciferase reporter plasmids for an additional 24 h. For experiments in which Notch activity was induced by Dll4, transfected HUVEC were replated onto Dll4-coated dishes 6 h after plasmid transfections. Luciferase activity was measured after an additional 24 h using the Dual-Luciferase Reporter Assay System (Promega) and a Synergy HT microplate reader. Luciferase reporter activity was normalized to the internal *Renilla* luciferase control and is expressed relative to control treatment.

### ChIP-qPCR

ChIP was performed using the ChIP-IT express kit (Active Motif). Briefly, HUVEC transfected with ERG or control siRNA, and/or treated with Ang1, were crosslinked for 10 min with formaldehyde (to a final concentration of 1%). Chromatin was sheared for five cycles (30 s on, 30 s off) using a Bioruptor UCD-200 ultrasound sonicator (Diagenode), resulting in DNA fragments of 200–1,000 bp in size. Chromatin was immunoprecipitated with 2 μg antibody to ERG (sc-354X, Santa Cruz Biotechnology), or negative control rabbit IgG (PP64, Chemicon, Millipore). Immunoprecipitated DNA was then used as template for quantitative PCR using primers specific for genomic loci. Oligonucleotide sequences are listed in Supplementary Table 1.

### Bioinformatic analysis

ERG transcription factor motif discovery was performed using the JASPAR database (http://jaspar.genereg.net). Genome-wide ChIP-Seq data for H3K27ac, H3K4me1 and H3K4me3 histone modifications, DNase I hypersensitivity, RNA polymerase II occupancy in HUVEC and phyloP sequence conservation (plotted as conservation scores between −5 and +5) based on Multiz alignment analysis of 100 vertebrate species were obtained from the Broad Institute and publicly available from the ENCODE Consortium. Tracks were visualized using the UCSC Genome Browser database (https://genome.ucsc.edu/index.html).

### Statistical analysis

No statistical methods were used to predetermine the sample size. No randomization was applied as all mice used were genetically defined, inbred mice. No blinding was used and no animals were excluded from analysis. Sample sizes were selected on the basis of previous experiments. All results presented in this study are representative of at least three independent experiments to guarantee reproducibility of findings. ‘*n*’ represents the number of biological replicates unless otherwise stated. Data are shown as the mean±s.e.m. Statistical significance was determined by two-tailed Student’s *t* test, using Prism 6.0 (Graph Pad). Differences were considered statistically significant with a *P* value<0.05.

### Data availability

The data that support the findings of this study are available within the article, its Supplementary Information files and from the corresponding author on reasonable request.

## Additional information

**How to cite this article:** Shah, A. V. *et al*. The endothelial transcription factor ERG mediates Angiopoietin-1-dependent control of Notch signalling and vascular stability. *Nat. Commun.* **8,** 16002 doi: 10.1038/ncomms16002 (2017).

**Publisher’s note:** Springer Nature remains neutral with regard to jurisdictional claims in published maps and institutional affiliations.

## Supplementary Material

Supplementary Information

Supplementary Movie 1

Supplementary Movie 2
