# Supplementary material for: The endothelial transcription factor ERG mediates Angiopoietin-1-dependent control of Notch signalling and vascular stability
Source: Nat Commun. 2017 Jul 11;8:16002. doi: 10.1038/ncomms16002 (PMC5508205; doi:10.1038/ncomms16002)
Supplement: Supplementary Information [file ncomms16002-s1.pdf]

Type of file: PDF
Title of file for HTML: Supplementary Information
Description: Supplementary Figures and Supplementary Table.

Type of file: AVI
Title of file for HTML: Supplementary Movie 1
Description: **Representative 3D reconstruction of confocal Z-stack images of skin samples from Erg$^{fl/+}$ mice, related to Figure 5:** FITC-dextran was injected through the tail vein of Erg$^{fl/+}$ mice to assess vascular permeability. Movie shows extravasation of FITC-dextran (green) from blood vessels stained for isolectin B4 (IB4, purple) and ERG (white).

Type of file: AVI
Title of file for HTML: Supplementary Movie 2
Description: **Representative 3D reconstruction of confocal Z-stack images of skin samples from Erg$^{cEC\text{-}het}$ mice, related to Figure 5:** FITC-dextran was injected through the tail vein of Erg$^{cEC\text{-}het}$ mice to assess vascular permeability. Movie shows extravasation of FITC-dextran (green) from blood vessels stained for isolectin B4 (IB4, purple) and ERG (white). In vivo deletion of endothelial ERG increases basal vascular permeability in established vessels.

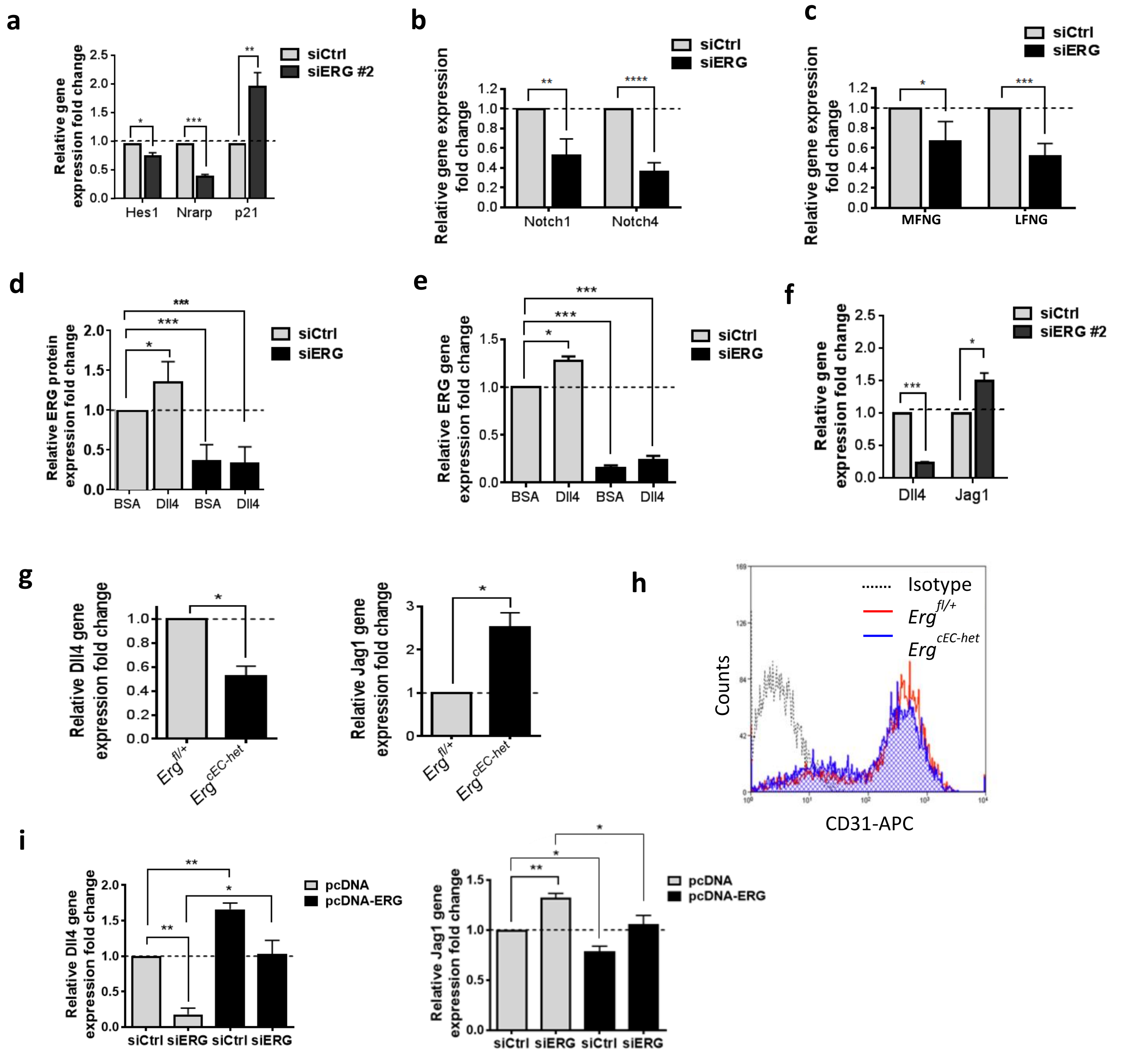

Supplementary Figure 1 ERG regulates multiple Notch related proteins and ERG overexpression is able to normalize the expression of Dll4 and Jag1 in siERG-treated HUVEC

(**a**) qPCR analysis of Notch effector and target gene expression following transfection of HUVEC with a 2nd siRNA targeting exon7 of ERG (siERG#2) (n=3). qPCR analysis of (**b**) Notch1 and Notch4 expression and of (**c**) Manic Fringe (MFNG) and Lunatic Fringe (LFNG) gene expression in siCtrl and siERG-treated HUVEC (n=4). (**d**) Western blot quantification of ERG expression in siCtrl and siERG-transfected HUVEC plated onto control BSA or Dll4 (n=4). (**e**) qPCR analysis of ERG mRNA expression in siCtrl and siERG-transfected HUVEC plated onto BSA or Dll4 (n=4). (**f**) qPCR analysis of Dll4 and Jag1 gene expression following transfection of HUVEC with a 2nd siRNA targeting exon7 of ERG (siERG#2) (n=3). (**g**) mRNA expression of Dll4 and Jag1 in primary $Erg^{cEC\text{-}het}$ mouse lung EC compared to control $Erg^{fl/+}$. *Hprt* was used as the reference gene (n=6). (**h**) Flow cytometric analysis for the endothelial marker CD31 in positively selected isolated lung cells from $Erg^{fl/+}$ and $Erg^{cEC\text{-}het}$ mice (n=4). (**i**) qPCR analysis of Dll4 and Jag1 gene expression in siCtrl and siERG HUVEC transfected with control pcDNA or pcDNA-ERG plasmid (n=3). All graphical data are mean ± s.e.m, *P < 0.05, **P < 0.01, ***P < 0.001, Student's t-test.

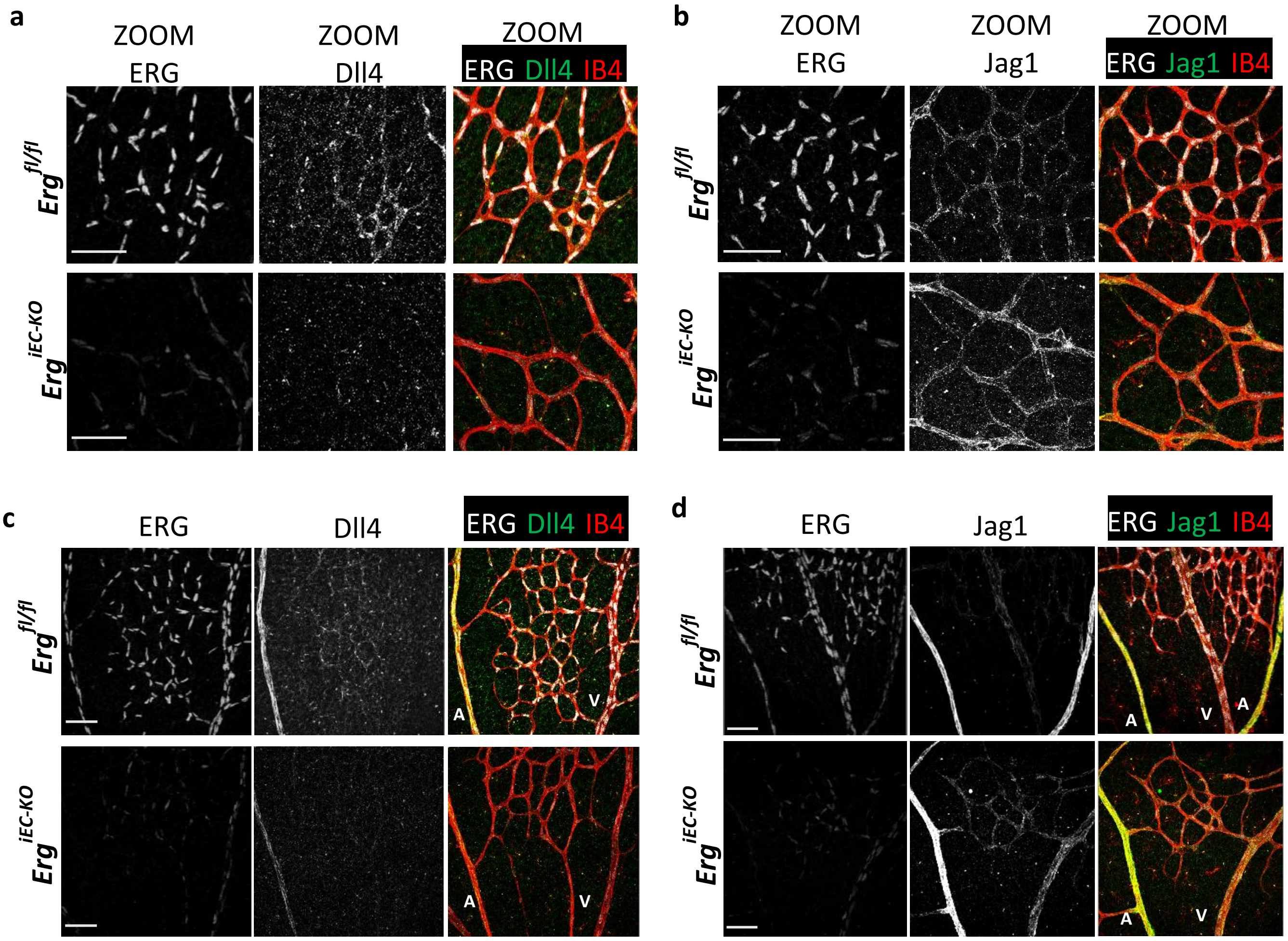

Supplementary Figure 2 ERG controls Dll4 and Jag1 expression in the remodelling plexus of the mouse retina
Zoom of (**a**) Fig. 3a and (**b**) Fig. 3b showing ERG and Dll4/Jag1 staining as individual channels and merged with isolectin B4 (IB4: red, ERG: white, Dll4/Jag1: green). Representative low magnification images of (**c**) Dll4 and (**d**) Jag1 staining of postnatal day 6 retinal vessels in the stable plexus of $Erg^{fl/fl}$ and $Erg^{iEC\text{-}KO}$ mice. Retinas are co-stained for ERG (white) and isolectin B4 (IB4: red). Scale bar, 70 µm. Arteries (A) and veins (V) are indicated.

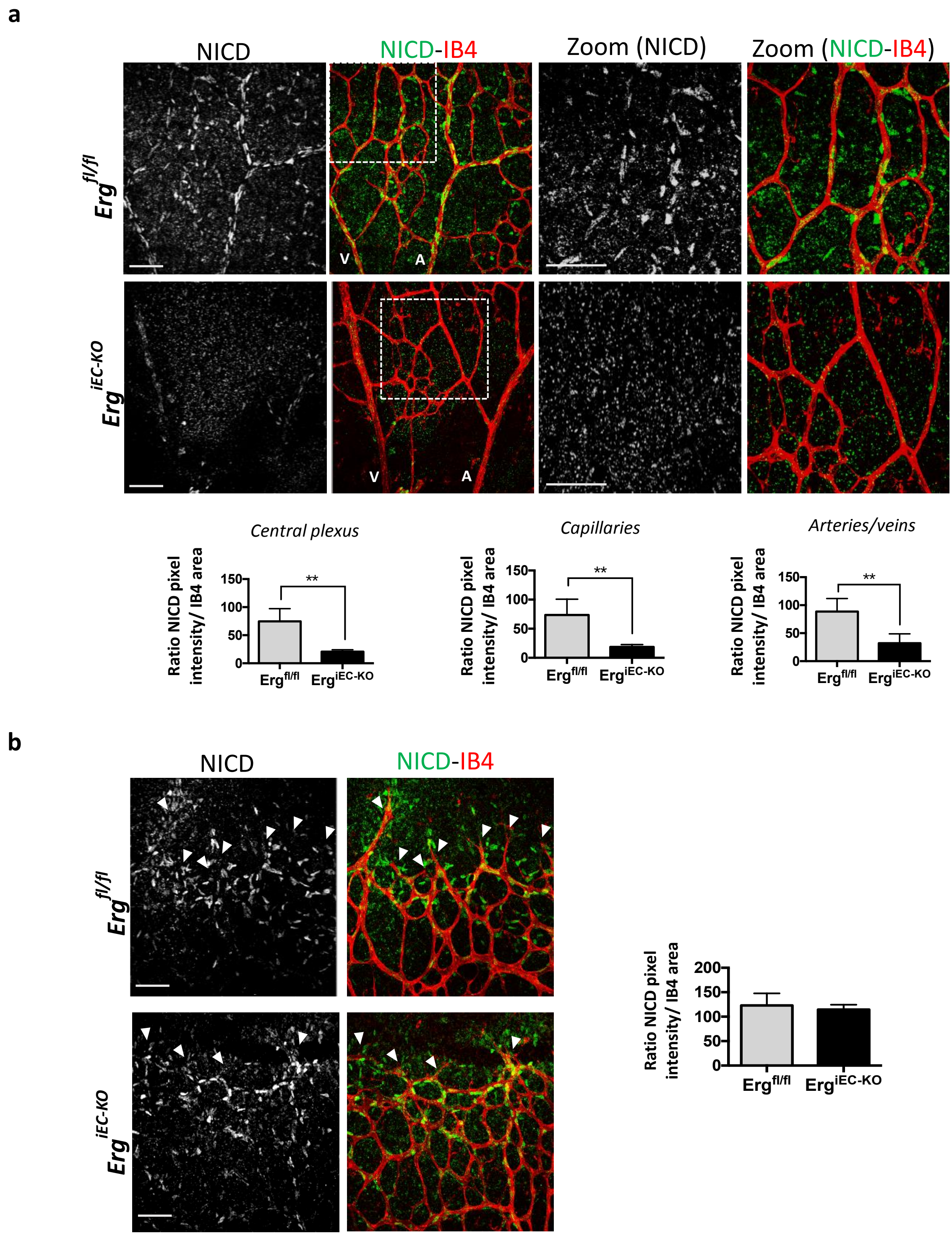

Supplementary Figure 3 ERG differentially controls NICD levels in the angiogenic front versus vascular remodelling plexus of the mouse retina

Representative images and quantification of NICD staining (green) of postnatal day 6 retinal vessels in the (**a**) stable plexus and (**b**) angiogenic front from $Erg^{fl/fl}$ and $Erg^{iEC\text{-}KO}$ mice. Retinas are co-stained for isolectin B4 (IB4, red). Quantification represents the ratio between the sum of pixel intensity and isolectin B4 area. Only NICD signal touching isolectin B4 signal was quantified (n=4 fields per mouse, n=3 mice per genotype). Scale bar, 70 µm. Arteries (A) and veins (V) are indicated. Arrowheads highlight tip cells at the angiogenic front. All graphical data are mean ± s.e.m, **P < 0.01, Student's t-test.

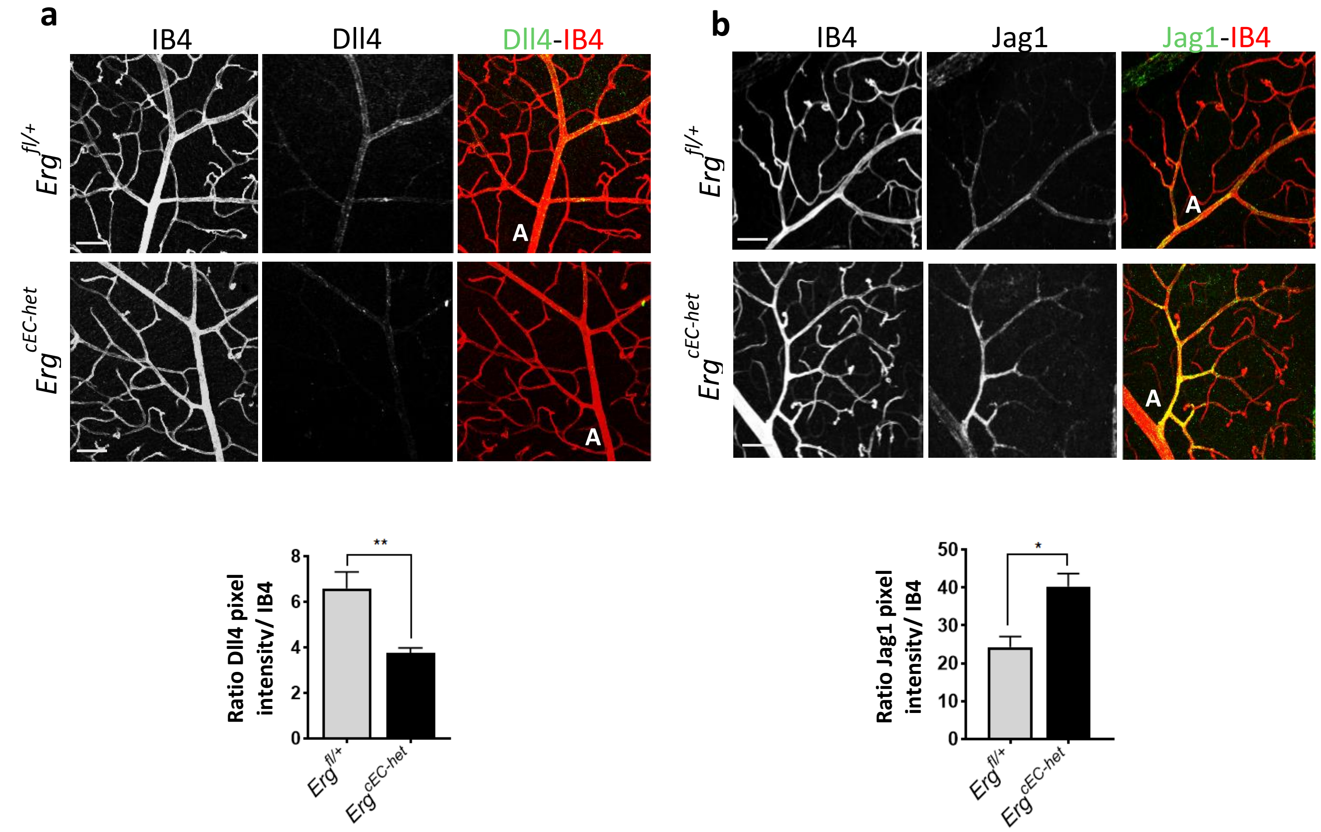

Supplementary Figure 4 ***In vivo*** **deletion of ERG downregulates Dll4 and upregulates Jagged 1 expression in established vessels**

Representative images and quantification of (**a**) Dll4 (green) and (**b**) Jag1 (green) staining of adult retina from 9 weeks-old *Erg*$^{cEC\text{-}het}$ and *Erg*$^{fl/+}$ mice (n=4). Retinas are co-stained for isolectin B4 (IB4, red). Scale bar, 70 µm. Quantification represents the ratio between the sum of pixel intensity and isolectin B4 area (n=4). All graphical data are mean ± s.e.m, *P < 0.05, **P < 0.01, ***P < 0.001, Student's t-test.

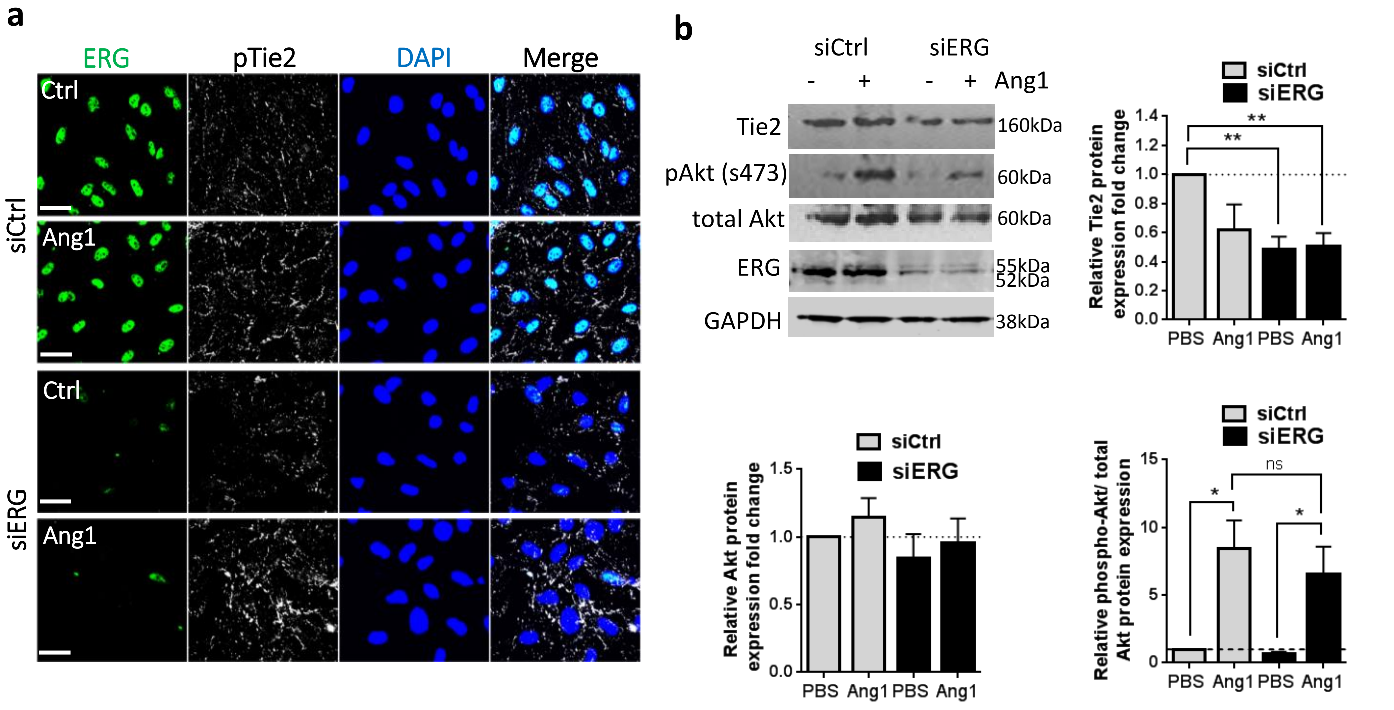

Supplementary Figure 5 Ang1 induction of Tie2 phosphorylation at cell-cell contacts is still active in ERG silenced cells

(**a**) ERG (green), phospho-Tie2 (pTie2; white), DAPI (blue) staining of control (siCtrl) and ERG-silenced (siERG) HUVEC treated in the present or absence of Ang1 (250 ng/ml for 30min). Scale bar, 30 µm. (**b**) Representative western blot and quantification of Tie2 expression and Akt phosphorylation (at serine 473) in extracts of siCtrl and siERG HUVEC treated in the presence or absence of Ang1 (n=4). Akt activity represents the ratio of phospho-Akt to total Akt. Data are mean ± s.e.m, *P < 0.05, **P < 0.01, ***P < 0.001, Student's t-test.

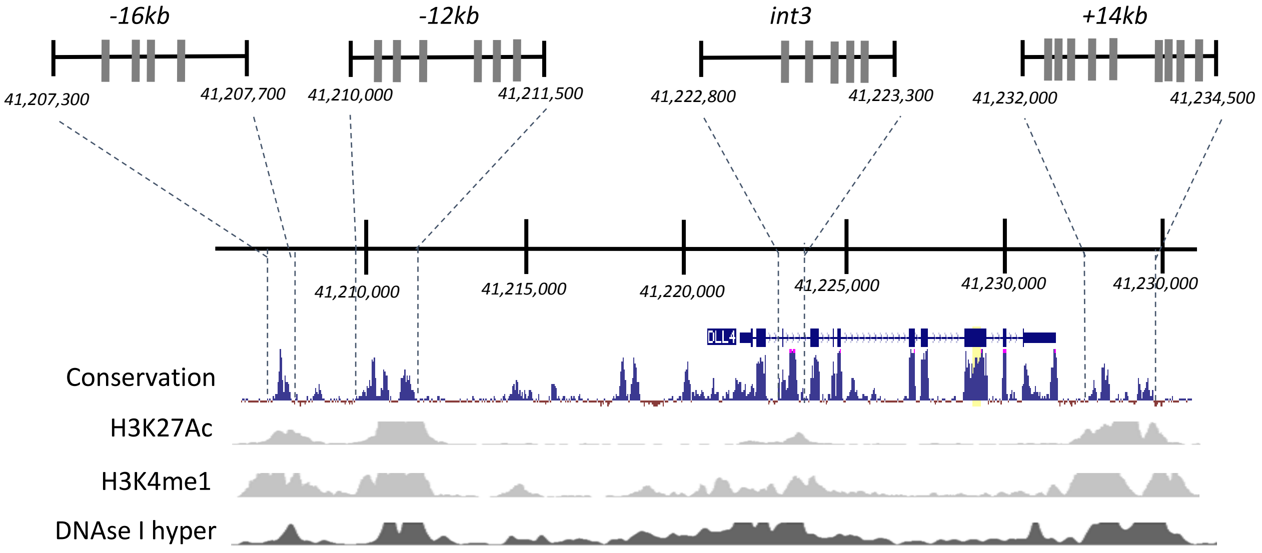

Supplementary Figure 6 ERG bound genomic regions at the Dll4 locus

Conserved ERG DNA binding motifs (grey bars) within the putative enhancers in the distal landscape surrounding the Dll4 locus. ENCODE sequence conservation between 100 vertebrates is shown across this region. ENCODE ChIP-seq data profiles for H3K27Ac, H3K4me1 and DNAse I hypersensitivity in HUVEC indicate open chromatin and putative active enhancers. Numbers above gene tracks indicate genomic location.

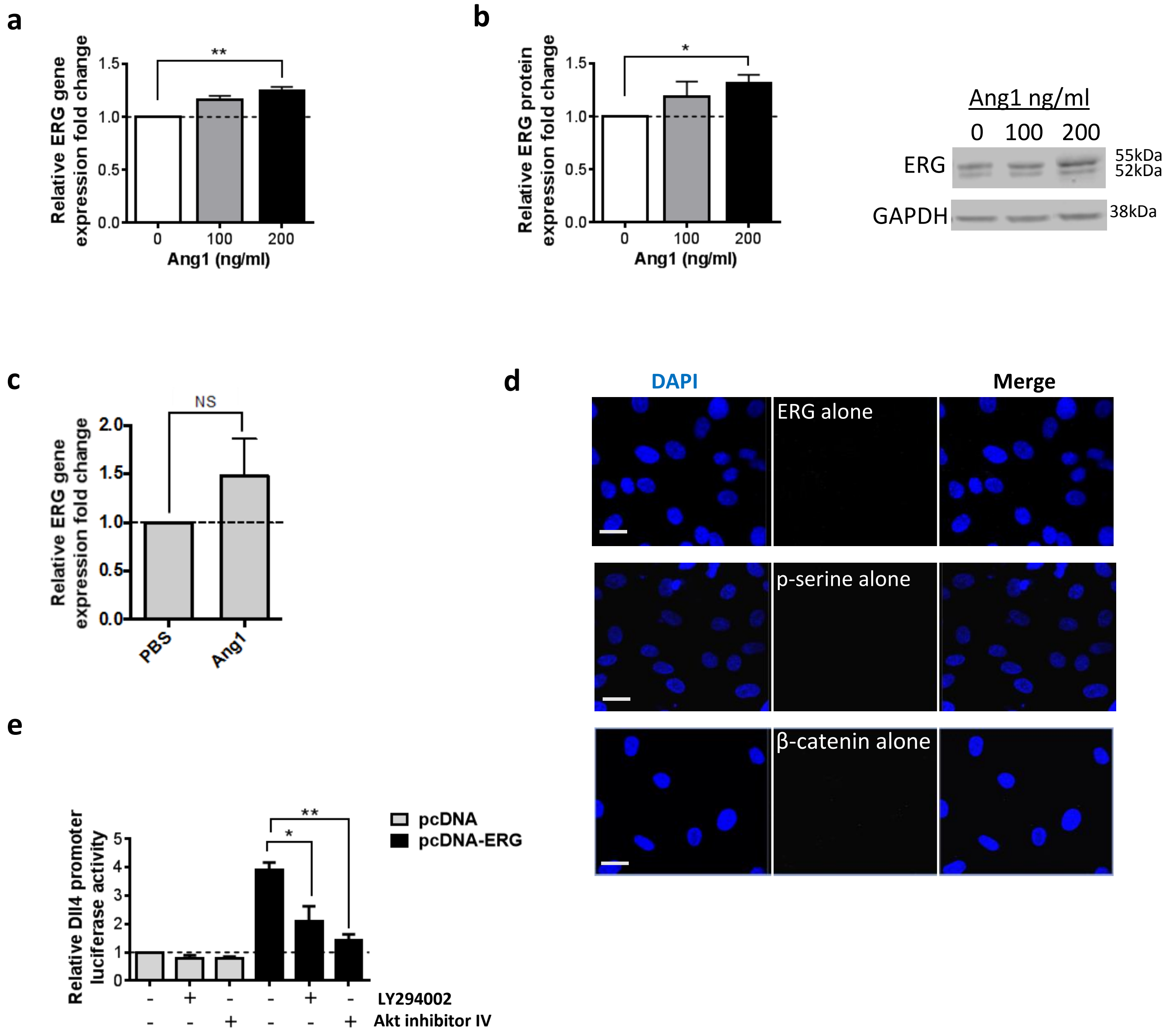

Supplementary Figure 7 Ang1 upregulates ERG expression *in vitro*

(**a**) qPCR and (**b**) western blot analysis of ERG expression in extracts of control and ERG-deficient cells treated with Ang1 at 0, 100, 200 ng/ml for 6 h (n=3). (**c**) qPCR analysis of ERG expression in extracts of skin samples from control *Erg*$^{fl/+}$ mice treated with intradermal injection of PBS or Ang1 (50 ng) for 1h (n=3). (**d**) Proximity ligation assay (PLA) analysis using rabbit anti-ERG, mouse anti-phospho-serine or mouse anti-β-catenin antibodies alone as controls. Scale bar, 20 µm. (**e**) Luciferase activity was measured in HUVEC co-transfected with an empty vector control or ERG cDNA expression plasmid (pcDNA-ERG) and the pGl4-Dll4 luciferase construct and treated in the presence or absence of LY294002 or Akt inhibitor IV (n=4). All graphical data are mean ± s.e.m, *P < 0.05, **P < 0.01, ***P < 0.001, Student's t-test.

a

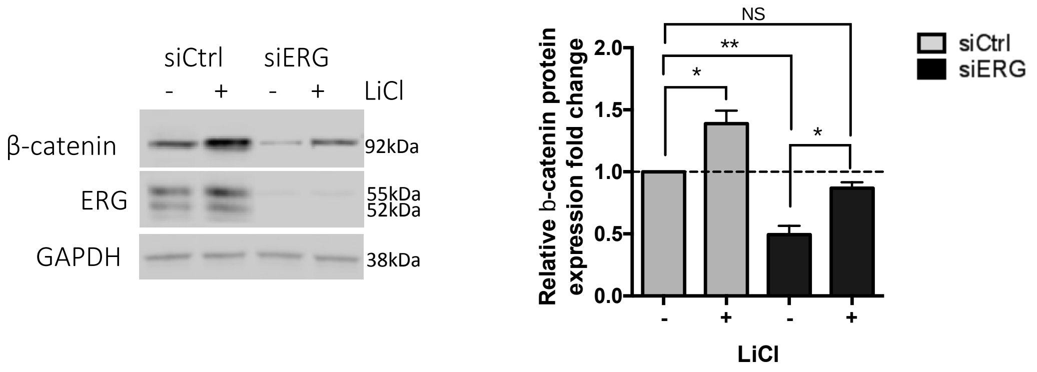

b

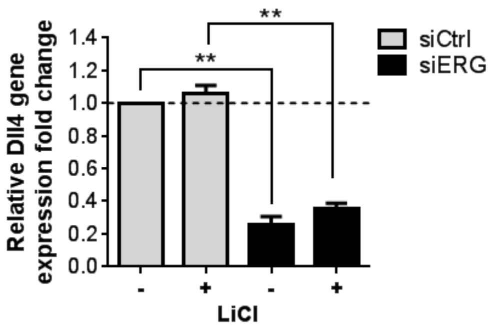

Supplementary Figure 8 Stabilisation of β-catenin by LiCl treatment in ERG deficient EC has no effect of Dll4 expression

(**a**) Western blot analysis and quantification of β-catenin expression in LiCl treated siCtrl and siERG HUVEC (n=3). (**b**) qPCR analysis of Dll4 expression in LiCl treated siCtrl and siERG HUVEC (n=3). Data are mean ± s.e.m, *P < 0.05, **P < 0.01, ***P < 0.001, Student's t-test.

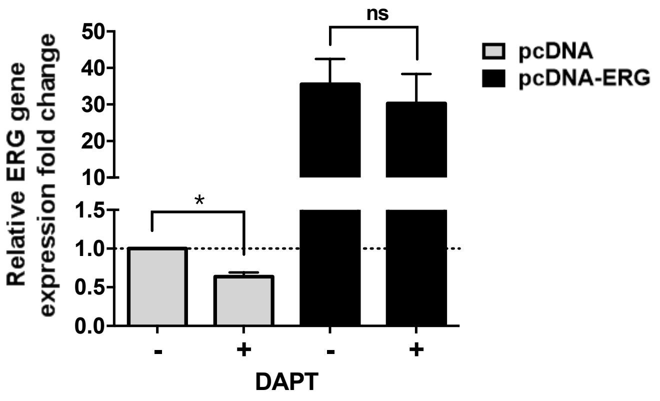

Supplementary Figure 9 Confirmation of ERG overexpression in HUVEC transfected with pcDNA-ERG expression plasmid

ERG mRNA expression in HUVEC transfected with control pcDNA or pcDNA-ERG plasmid and treated in the presence or absence of the γ-secretase inhibitor DAPT (n=3). Data are mean ± s.e.m, *P < 0.05, **P < 0.01, ***P < 0.001, Student's t-test.

Fig. 1a

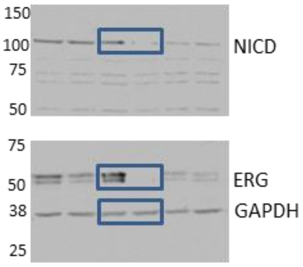

Fig. 1g

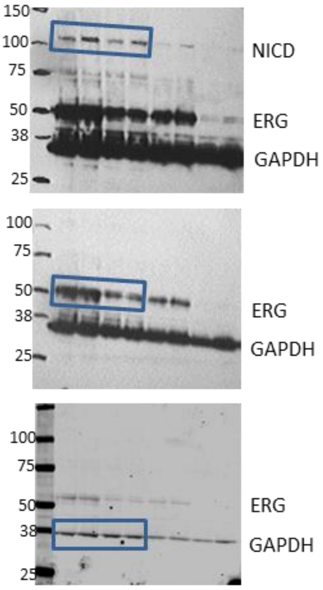

Fig. 2b

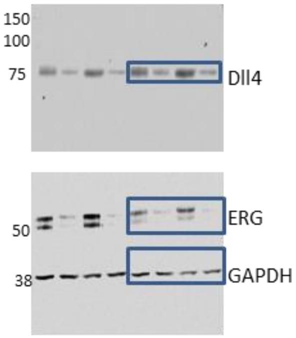

Fig. 2d

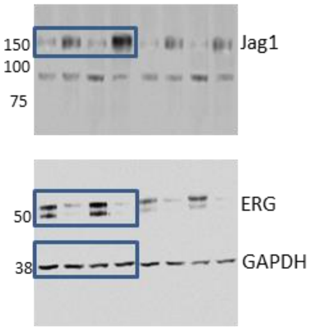

Fig. 4d

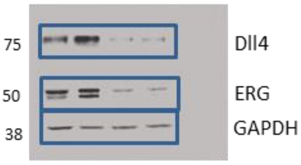

Fig. 4f

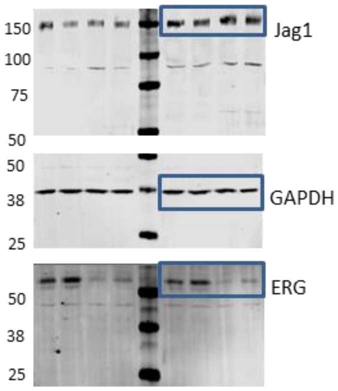

Fig. 7c

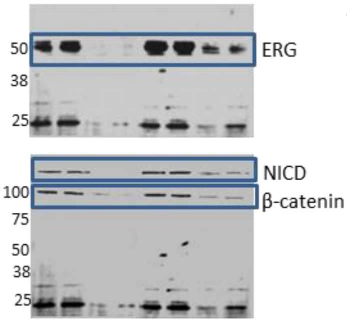

Supplementary Fig. 7b

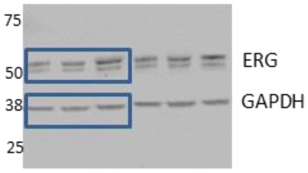

Supplementary Fig. 8a

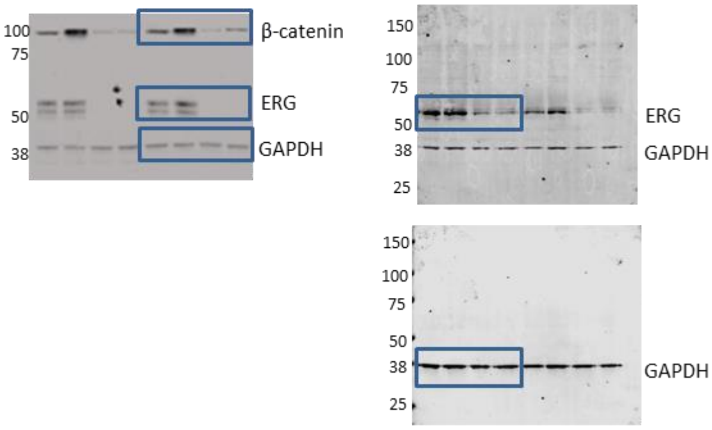

Supplementary Fig. 5b

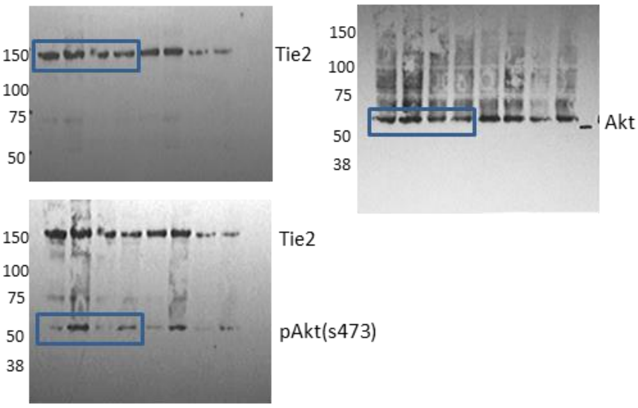

Supplementary Figure 10 Uncropped Western blotting images for figures

Supplementary Table 1 Oligonucleotides used in this study

Primers		Oligonucleotide Sequences
DLL4 (human)	Forward	5’- CTGGCCGACGCTGTGAGGTG -3’
	Reverse	5’- GGCAAGCCCACGGGGAACTC -3’
Dll4 (mouse)	Forward	5’- TTTGCTCTCCCAGGGACTCT -3’
	Reverse	5’- AGGCTCCTGCCTTATACCTCT -3’
Dll4 (human) promoter (cloning)	Forward (Nhe1)	5’-ACGTGCTAGCGGGCCAGAACCTCATTACC-3’
	Reverse (Hind III)	5’-ACGTAAGCTTCGCCGCTACTGAAACCTG-3’
DLL4 -16 enhancer (ChIP)	Forward	5’- TCATTCAAAAGCTCGGCCCT -3’
	Reverse	5’- TGATGCCCTGCGCTAGATTT -3’
DLL4 -12 enhancer (ChIP)	Forward	5’- TCCCACGCCCTCTATGAGTA -3’
	Reverse	5’- GCAGGACATCACAGCGTTTC -3’
DLL4 R1 promoter (ChIP)	Forward	5’- GGGAACACGAGGCCAAGAG -3’
	Reverse	5’- CTGTCTAATCCTGGGGCTGC -3’
DLL4 int3 enhancer (ChIP)	Forward	5’- GTTTCCTGCGGGTTATTTTT -3’
	Reverse	5’- CTTTCCAAAGGAGCGGAAT -3’
DLL4 +14 enhancer (ChIP)	Forward	5’- GGGGTTGTGCAGAAGGAGAA -3’
	Reverse	5’- TTTTCCCTACCCCCTGACCA -3’
DLL4 Ctrl exon 11 (ChIP)	Forward	5’- CTCAGGGCAGTGTGTTGGAA -3’
	Reverse	5’- CTCGAGGTTGTGGAGATGGG -3’
ERG (human)	Forward	5’- GGAGTGGGCGGTGAAAGA -3’
	Reverse	5’- AAGGATGTCGGCGTTGTAGC -3’
ERG (mouse)	Forward	5’- CCGGATACTGTGGGGATGAG -3’
	Reverse	5’- TCTGCGCTCATTTGTGGTCA -3’
GAPDH (human)	Forward	5’- CAAGGTCATCCATGACAACTTTG -3’
	Reverse	5’- GGGCCATCCACAGTCTTCTG -3’
HES1 (human)	Forward	5’- AATTCCTCGTCCCCGGTGGCT -3’
	Reverse	5’- CTTGGAATGCCGCGAGCTATCTT -3’
HEY1 (human)	Forward	5’- TCGGCTCTAGGTTCCATGTCCCC -3’
	Reverse	5’- AGCTTAGCAGATCCCTGCTTCTCAA -3’
Hprt (mouse)	Forward	5’- GTTAAGCAGTACAGCCCCAAAATG -3’
	Reverse	5’- TCAAGGGCATATCCAACAACAAAC -3’
JAG1 (human)	Forward	5’- GTTTCGCCTGGCCGAGGTCC -3’
	Reverse	5’- GTGGGCAACGCCCGTGTTCT -3’
Jag1 (mouse)	Forward	5’- CTGCTTGAATGGGGGTCACT -3’
	Reverse	5’- GCAGCTGTCAATCACTTCGC -3’
JAG1 R1	Forward	5’- GAGCACGCCCTCTCATGAAT -3’
	Reverse	5’- GCCGCAGGTAACACAATGAC -3’
JAG1 R2	Forward	5’- GGGTGGAAGGAAGATGGGTG -3’
	Reverse	5’- AGTGCACCCCATTAGAGCAC -3’
JAG1 R3	Forward	5’- ACTCCATGGCGGTTACCTTG -3’
	Reverse	5’- CGGCTGCCAACACAATTACC -3’
JAG1 Ctrl 3’UTR	Forward	5’- CCTGACAGAGGGATGGAGGA -3’
	Reverse	5’- AGGGAATCAAGGCTCCCCTA -3’

**Primers**		**Oligonucleotide Sequences**
LFNG (human)	Forward	5’- GCCACAAGGAGATGACGTT-3’
	Reverse	5’- CCGAGCAGTTTGTGATGACC -3’
MNFG (human)	Forward	5’- AACAGGTGACAAGGTCCCAC -3’
	Reverse	5’- GTCGAACTCAGCAGCCATCT -3’
NOTCH1 (human)	Forward	5’- ACCAATACAACCCTCTGCGG -3’
	Reverse	5’- AGCTCATCATCTGGGACAGG -3’
NOTCH4 (human)	Forward	5’- CTGCAGTGGACGCTCGCACA -3’
	Reverse	5’- CTGGCCCCCACGTCTGCTTG -3’
NRARP (human)	Forward	5’- GCGCTGCACCAGTCGGTCAT -3’
	Reverse	5’- GCCGCGTACTTCGCCTTGGT -3’
